# Mean Annual Precipitation Explains Spatiotemporal Patterns of Cenozoic Mammal Beta Diversity and Latitudinal Diversity Gradients in North America

**DOI:** 10.1371/journal.pone.0106499

**Published:** 2014-09-09

**Authors:** Danielle Fraser, Christopher Hassall, Root Gorelick, Natalia Rybczynski

**Affiliations:** 1 Department of Biology, Carleton University, Ottawa, Ontario, Canada; 2 Palaeobiology, Canadian Museum of Nature, Ottawa, Ontario, Canada; 3 School of Biology, University of Leeds, Leeds, United Kingdom; 4 Department of Mathematics and Statistics, Carleton University, Ottawa, Ontario, Canada; 5 Institute of Interdisciplinary Studies, Carleton University, Ottawa, Ontario Canada; Monash University, Australia

## Abstract

Spatial diversity patterns are thought to be driven by climate-mediated processes. However, temporal patterns of community composition remain poorly studied. We provide two complementary analyses of North American mammal diversity, using (i) a paleontological dataset (2077 localities with 2493 taxon occurrences) spanning 21 discrete subdivisions of the Cenozoic based on North American Land Mammal Ages (36 Ma – present), and (ii) climate space model predictions for 744 extant mammals under eight scenarios of future climate change. Spatial variation in fossil mammal community structure (β diversity) is highest at intermediate values of continental mean annual precipitation (MAP) estimated from paleosols (∼450 mm/year) and declines under both wetter and drier conditions, reflecting diversity patterns of modern mammals. Latitudinal gradients in community change (latitudinal turnover gradients, aka LTGs) increase in strength through the Cenozoic, but also show a cyclical pattern that is significantly explained by MAP. In general, LTGs are weakest when continental MAP is highest, similar to modern tropical ecosystems in which latitudinal diversity gradients are weak or undetectable. Projections under modeled climate change show no substantial change in β diversity or LTG strength for North American mammals. Our results suggest that similar climate-mediated mechanisms might drive spatial and temporal patterns of community composition in both fossil and extant mammals. We also provide empirical evidence that the ecological processes on which climate space models are based are insufficient for accurately forecasting long-term mammalian response to anthropogenic climate change and inclusion of historical parameters may be essential.

## Introduction

Terrestrial species from all major taxonomic groups show dramatic changes in richness and diversity across the landscape [1]. One of the fundamental goals in ecology is therefore to ascertain why there are more species in some places than in others. A satisfactory answer would identify and disentangle the drivers of biodiversity at all spatial scales, from the microhabitat to the globe, as well as explain changes through time. Attempts to provide such an answer have produced many studies of species richness patterns and community composition in extant organisms [1]–[8]. Prime examples are the numerous studies of latitudinal richness gradients (LRGs), which have been observed in many terrestrial groups including angiosperms, birds, mammals, insects and other invertebrates. The best supported hypotheses show that richness declines toward the poles in correlation with reductions in precipitation, temperature, and net primary productivity [9]. Correlation of global climate with animal richness over the past 65 Ma, specifically a decline in richness as climates cooled, similarly supports a link between diversity and climate [10]–[12]. However, of the spatial and temporal dimensions of diversity, spatial patterns of community differences (“β diversity”) are infrequently studied despite considerable variation on both local and regional scales [2], [13], [14] and their influential role in the structuring of continental-scale richness patterns including LRGs [3], [4].

β diversity has been defined most broadly as the differentiation in community composition (i.e. the species that make up the community) among regions or along environmental gradients [15]. Similar to LRGs, β diversity generally declines from the tropics to the poles in correlation with climate [2]. However, temporal changes in β diversity remain poorly studied despite their potential power for illuminating the drivers of past and present richness patterns and importance in modern conservation [16]–[18]. This study therefore tests the hypothesis that climatic influences on mammalian β diversity apply equally to temporal patterns, i.e. that the underlying ecological processes are “ergodic” (dynamic processes that are the same in both time and space).

The mid to late Cenozoic (36 Ma to present) has been a time of dramatic mammalian diversity change, shaped in part by the transition from the productive ice-free ecosystems of the early to mid Cenozoic to the more temperate glaciated ecosystems of the late Cenozoic. Under these changing climatic conditions, mammalian communities show dramatic reductions in richness, changes in community composition, and morphology [10], [19]–[24]. The most dramatic changes occurred at high latitudes, where ecosystems transitioned from *Metasequoia* forests during the early to mid Cenozoic [25], [26] to boreal-type forests during the later Cenozoic and to modern tundra [27]. Associated with Cenozoic climate change, were changes in latitudinal climate gradients; overall, the intensity of latitudinal climate gradients increased toward the present, reflecting disproportionate polar cooling due to the formation of permanent Arctic glaciation [28], [29]. We therefore predict that latitudinal diversity gradients increased in strength under cooler, less productive environmental conditions just as modern LRGs are steeper in temperate than in tropical regions. Further, we predict that β diversity declined under cooler, less productive environmental conditions just as modern β diversity declines toward the poles [2], [7].

Quaternary (2.6 Ma to present) climates have been cool relative to the majority of the late Cenozoic. Recently, however, high latitudes have experienced disproportionate increases in annual temperature (up to 2°C to date), increases in plant primary productivity, and loss of large areas of perennial ice under anthropogenic global warming [30]. Flora and fauna have responded through shifts in phenology [31], *in situ* evolution [32], and, in some cases, extinction [33]. However, perhaps the most often recorded response is the climatically-correlated pattern of extirpations and colonization that manifest as shifts in the location of a species’ geographic range. Distributional studies over ecological timescales (<100 yrs) have recorded dramatic pole-ward range shifts and expansions for a wide range of terrestrial taxa in response to northern warming [34], [35]. Projections (i.e. Special Report on Emissions Scenarios) for the next 100 years predict levels of global warming similar to the middle Miocene (+6°C) − a time of reduced or absent perennial Arctic glaciation [36], [37] − or warmer (+11°C for the most extreme case; Table S1). We therefore expect continued range expansion, extinction, evolution, and community level changes among North American animals and plants.

A common approach to predicting the long-term outcomes of climate change for terrestrial organisms is climate space modeling (CSM). CSMs use distributional information and climate data to project species ranges into the future, usually under the assumption of no evolution and without adjustment for dispersal differences among species [38]–[40]. Rapid evolutionary changes on very short timescales and high degrees of variation in dispersal ability under climate change have been observed across a wide range of organisms [34], [39], [41], therefore CSMs are unlikely to generate accurate forecasts of climate change response. The fossil record, which encompasses many disparate environments and climates, might serve as record of a natural experiment by which ecological hypotheses can be tested in the temporal dimension. Fossil collections are a rich historical record of response to various climatic events that can be incorporated into predictive models, and mammals, in particular, are an excellent group for testing the generality of ecological hypotheses because they have an extensive Cenozoic fossil record. However, studies of extinct organisms have focused largely on richness [12], [22], [23], [42], [43] or morphology [44], with limited focus on community composition [20], [22]. Because changes in biological communities are not always associated with changes in richness, spatiotemporal patterns of community composition may be better indicators of climate change response [13], [18].

We propose that integrating the study of fossil, modern, and projected spatiotemporal patterns of community composition i) allows for the testing of ecological principles in the temporal dimension, ii) provides the most complete picture of diversity responses to climate change, and iii) enables evaluation of the performance of commonly employed CSMs. Our approach of combining the study of fossil, modern, and projected diversity patterns provides novel insights into the ecological and evolutionary processes that drive continental patterns of biodiversity in space and time.

## Methods

### Data collection and preparation

We downloaded occurrences for modern North American mammals from NatureServe Canada. The extant mammal dataset included 744 species after the exclusion of a small number of unreadable or corrupted files [45]. We restricted our study of fossil mammals to the late Eocene through Pleistocene, thus avoiding the confounding effects of the early Paleogene mammal radiation. We partitioned the fossil mammal occurrence data by North American Land Mammal Age (NALMA) subdivisions because they delineate relatively temporally stable community assemblages and allowed us to obtain a nearly continuous sequence of mammal community change without large intervening gaps. Using NALMA subdivisions leads to time averaging of mammal communities and to differences in sampling (i.e. intensity, geographic coverage etc.) among time periods. However, we use a statistical approach to reduce these biases, described below. We based the dates for all NALMA subdivisions on Woodburne (2004). Further, we combined data for the entire Clarendonian and excluded for the Whitneyan, late Late Hemphillian, and early Chadronian due to poor sampling (Table 1).

**Table 1:** Summary of sampled North American Land Mammal Age (NALMA) subdivisions.

Epoch	NALMA subdivision	Age Range (Ma)	Midpoint Age (M)	Number of species	Number of fossil localities	Area (km^2^)
Pleistocene	Rancholabrean	0.25–0.011	0.1305	222	180	176615.9
Pliocene	Irvingtonian II	0.85–0.25	0.55	189	94	144745.5
Pliocene	Irvingtonian I	1.72–0.85	1.285	102	37	60361.4
Pliocene	Blancan V	2.5–1.72	2.11	165	130	125042.6
Pliocene	Blancan III	4.1–2.5	3.3	183	163	122839.5
Pliocene	Blancan I	4.9–4.1	4.5	85	66	140433.4
Miocene	Early late Hemphillian	6.7–5.9	6.3	68	46	20108.2
Miocene	Late early Hemphillian	7.5–6.7	7.1	63	55	29446.7
Miocene	Early early Hemphillian	9–7.5	8.25	65	47	31455.8
Miocene	Clarendonian	12.5–9	10.75	104	90	36139.8
Miocene	Late Barstovian	14.8–12.5	13.6	195	194	33789.1
Miocene	Early Barstovian	15.9–14.8	15.5	150	168	51753.3
Miocene	Late Hemingfordian	17.5–15.9	16.7	100	83	25478.4
Miocene	Early Hemingfordian	18.8–17.5	18.15	107	105	45531.3
Miocene	Late late Arikareean	19.5–18.8	19.15	108	123	38307.2
Oligocene/Miocene	Early late Arikareean	23.8–19.5	21.65	71	67	37892.2
Oligocene	Late early Arikareean	27.9–23.8	25.85	95	65	20927.8
Oligocene	Early early Arikareean	30–27.9	28.95	116	124	15382.3
Oligocene	Late Orellan	33.1–32	32.55	38	36	17725.7
Oligocene	Early Orellan	33.7–33.1	33.4	88	130	5579.8
Eocene	Middle Chadronian	35.7–34.7	35.3	88	37	10349.7

We downloaded fossil mammal occurrence data for the Eocene, Oligocene, Pliocene, and Pleistocene from the the Paleobiology Database using the Fossilworks Gateway (fossilworks.org) in July and August, 2012, using the group name ‘mammalia’ and the following parameters: time intervals = Cenozoic, region = North America, paleoenvironment = terrestrial (primary contributor: John Alroy; literature sources summarized in Appendix S1). We downloaded Miocene mammal occurrence data from the Miocene Mammal Mapping Project in March 2011 [46] using the NALMA subdivision as our search criterion. For all analyses, with the exception of the Miocene, we used paleolatitudes and paleolongitudes. We chose to use MIOMAP for the Miocene data because it is the most complete Miocene dataset. However, MIOMAP does not provide paleo-coordinates. Fortunately, there are only small differences between modern and Miocene latitudes for the downloaded localities. We removed all taxa with equivocal species identifications (e.g. *Equus* sp.) unless they were the only occurrence for a genus. We assumed all occurrences of open nomenclature (e.g. *Equus* cf. *simplicidens*) were correct identifications.

We did not use latitudinal grids for fossil or extant mammals as in previous studies of latitudinal richness gradients [1], [47] because our study is focused on community composition. We therefore do not need to clump localities by spatial proximity to employ rarefaction methods. In addition, the uneven spatial distribution of fossil localities makes the use of a grid method impractical. Instead, we created taxon-by-locality occurrence matrices for extant and fossil mammals at the species taxonomic level excluding *Homo sapiens* [20], [22]. In all cases, taxa and localities with fewer than two occurrences were removed from the dataset. Final numbers of localities and species are summarized in Table 1.

To make direct comparisons with modern mammals, we created occurrence matrices for extant mammals by pseudo fossil localities, which were generated using an iterative procedure in R with the maptools, sp, gpclib, ggplot2, rgeos, and MASS packages [48]–[54] (contact corresponding author for R code). To generate pseudo fossil localities and to ensure that we created pseudo fossil localities with the same spatial distributions as the fossil localities, we fit frequency distributions (normal, gamma, or β) to fossil localities for each NALMA subdivision (Fig. S1). We then generated point samples based on the frequency distributions and the number of fossil localities from which we created occurrence matrices (taxon-by-pseudo locality), repeating the procedure 100 times for each NALMA sub-age for a total of 2100 occurrence matrices. Fossil localities do not record the entire community and so show reduced richness compared to the actual communities (however, note that time averaging also increases richness at fossil localities). Further, most fossil localities, unless intensively screen washed, are biased against small species. Therefore, we also intentionally tested for the effects of sampling bias by removing 25%, 50%, and 75% of species from the extant mammal occurrence matrices for a total of 6300 occurrence matrices. Further, we tested for the effects of body mass bias by 25%, 50%, and 75% of species smaller than 5 kg for a total of 6300 occurrence matrices.

### Climate space models

To create climate space models, we sampled the ranges of extant North and South American mammals at a series of 5066 points corresponding to a 1° grid (which we only used to project mammal occurrences under climate change models, but not to calculate biodiversity). Due to the focus on North America, we omitted any species with southern hemisphere ranges that did not cross the equator (n = 602; Table S2). We also excluded rare species (present in <20 cells) for which accurate species distribution models could not be generated (n = 361), leaving 706 species for the climate change projections. We extracted mean annual and winter (December, January, February) temperature and mean annual precipitation data from Climate Wizard (www.climatewizard.org) for the period of 1951–2006 and the following SRES scenarios and time periods: B1 2050s, A1b 2050s, A1b 2080s, A2 2050s, and A2 2080s [55] (Table S1). Each of these projections is based on an ensemble of 16 global circulation models [56]. However, to ensure that we sampled a range of potential warming, we also extracted the ensemble lowest B1 2050s projection (hereafter “B1 2050s low”) and the ensemble highest A2 2080s projection (hereafter “A2 2080s high”). This gave a range of warming in North America from 1.49°C (B1 2050s low) to 6.78°C (A2 2080s high, see Table S1 for the full range).

We modeled species’ ranges with the BIOMOD package in R using generalized linear models, generalized boosted models, classification tree analysis, artificial neural networks, surface range envelopes, flexible discriminant analysis, multiple adaptive regression splines, and random forests [57] (contact corresponding author for R code). We then used these models to make consensus forecasts for each of the projections described above, as well as current climate to evaluate the performance of the models. We tested model performance using area under the receiver operating curve (AUC), true skill statistic (TSS), and proportion correct classification (PCC, Fig. S2). Species and generic presences were determined across the 1° latitude-longitude grid to give presence or absence in each location at each time and SRES scenario.

Using the projections described above, we created pseudo localities, as before. From this, we created occurrence matrices as described above. We repeated this process 100 times for each projection for a total of 16,800 occurrence matrices.

### Latitudinal turnover gradients (LTGs) and β diversity

We calculated β diversity as the change in mammalian communities across the North American landscape using multivariate dispersion and the Jaccard index for each NALMA sub-age, for modern mammals, and for the climate projections [58]. We calculated Euclidean distances from the centroid for localities using the R package vegan [59]. Larger distances from the centroid indicate greater spatial community turnover and thus higher β diversity. We did not regress the Jaccard index values against distance, as has been used for modern species [2] because we have found such an approach to be highly influenced by species-area relationships.

To estimate ancient, modern, and projected LTG strength for North American mammals, we calculated the amount of community change with latitude using detrended correspondence analysis (DCA; an ordination technique) in the vegan R package [59]. We used explained variance (R^2^; how much of the variation in community change is explained by latitude) as a measure of LTG strength [13]. High values of explained variance indicate strong LTGs [60]. We did not compute latitudinal richness gradients because sampling bias (e.g. loss of taxa, body mass bias) is too great (Fraser, D. unpub.).

### Sampling bias control

Although we have chosen methods that minimize the effects of sampling bias, we still used multiple methods to control for the non-independence of β diversity from the number of localities, the geographic area sampled, and the number of sampled taxa. We used three approaches. Firstly, we used a re-sampling approach wherein we sub-sampled (without replacement) each NALMA 100× using a standardized number of localities (thirty) and limited to localities occurring between 30°and 50° North latitude. We also re-sampled the extant mammal ranges under various conditions of bias (taxonomic bias through the removal of 25%, 50%, 75% of taxa and body mass bias where we removed 25%, 50%, and 75% of species with a body mass lower than 5 kg) as above to test for direct causality of sampling bias. We also used a method of detrending whereby we regressed LTG strength and β diversity against statistically significant sampling bias metrics and further analyzed the residuals from the model. Finally, we used multivariate linear models to simultaneously account for the model variance explained by sampling and biological phenomena. The last multivariate method is similar to [61] and [62] (also addressed in [63]) who combine the predictive properties of models of biodiversity change and taphonomic bias.

### Correlation with climate

We tested for correlations of β diversity and LTG strength with stable oxygen isotopes from benthic foraminifera (δ^18^O ‰) [64], [65], mean annual precipitation estimated from paleosols [66], number of localities, sampling area (km^2^), number of species, latitudinal range (degrees), and length of the sampled interval (Ma) of the fossil localities using generalized least squares and using an autocorrelation structure of order one (corAR1) to account for temporal autocorrelation in R [67], [68]. Best fit models were selected using automated model selection in the MuMIn R package [69] and the Akaike Information Criterion (ΔAIC).

## Results

**Figure 1:**
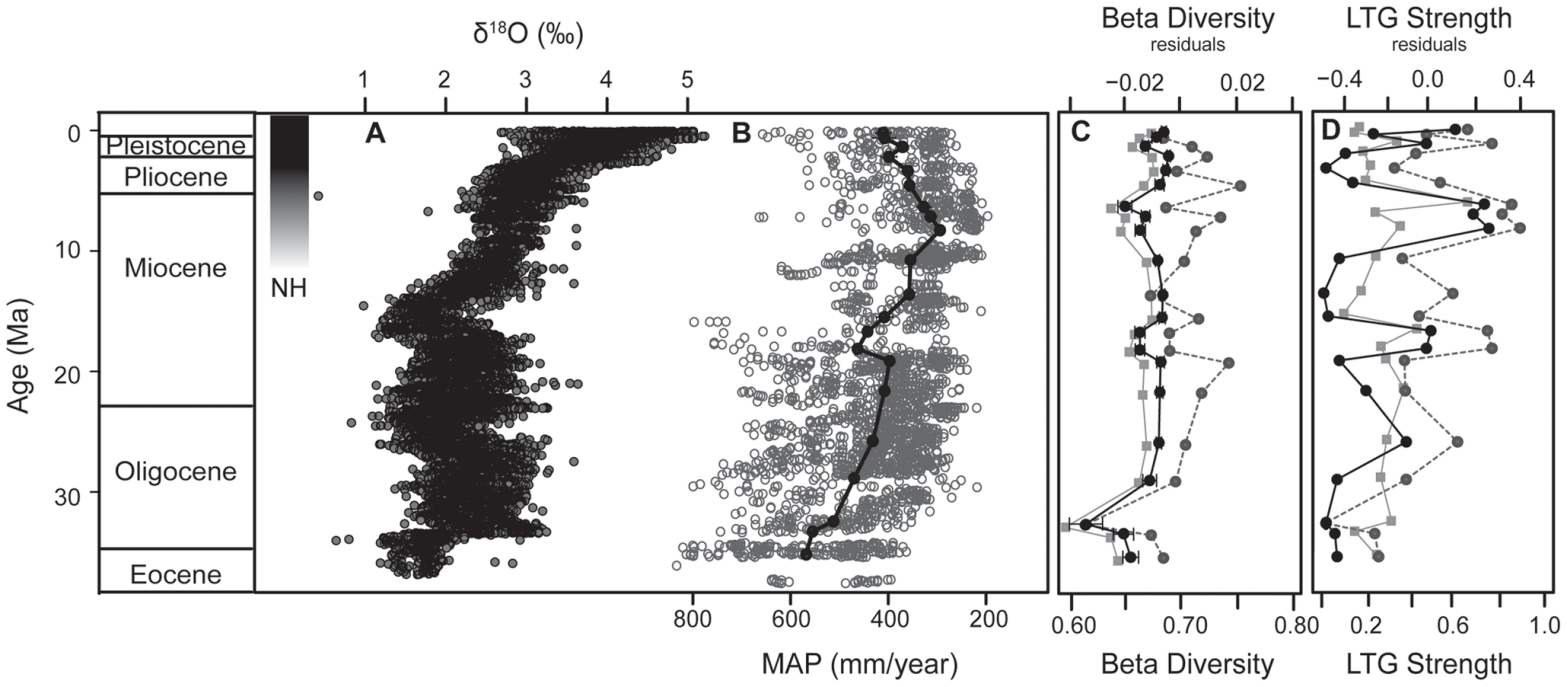
Mid to late Cenozoic trends of (A) δ^18^O (‰) from benthic foraminifera (Zachos et al. 2008), (B) mean annual precipitation estimated from paleosols (Retallack, 2007), (C) β diversity of North American mammal species measured using multivariate dispersion (average distance from the centroid), and (D) strength of latitudinal turnover gradients (LTGs) measured as gradient strength for North American fossil mammals. Black lines are raw values, gray lines are residuals from significant sampling bias predictors, and gray dashed lines are re-sampled. Standard errors for re-sampled data are too small to display.

**Figure 2:**
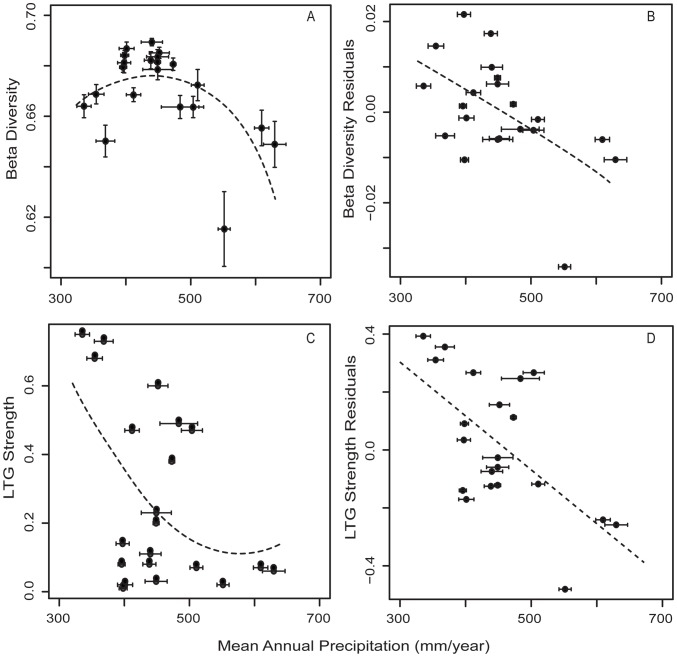
Relationship of mean annual precipitation estimated from paleosols (Retallack, 2007) with North American fossil mammal (A) raw β diversity (R^2^ = 0.43), (B) residual beta diversity (R^2^ = 0.26) and (C) raw latitudinal turnover gradient (LTG) strength (R^2^ = 0.25), and (D) residual LTG strength (R^2^ = 0.37).

Fossil mammal β diversity showed considerable variation with the warmest intervals (late Eocene, mid-late Oligocene, mid Miocene, and mid Pliocene), but showing generally higher β diversity than with cooler intervals (early Oligocene, late Miocene) (Fig. 1C). The best fit model includes mean annual precipitation (MAP squared), length of the NALMA subdivision, and number of taxa, which together accounts for 67% of model variance (Table 2). β diversity is statistically significant for all three predictors (p<0.05). Residual β diversity is significantly explained by MAP only (Table 2; Fig. 2B). Re-sampling did not alleviate the effects of sampling bias; re-sampled β diversity is significantly explained by MAP-squared, number of taxa, and NALMA subdivision length (Table 2). The remainder of the manuscript will discuss the results from the analyses of raw and residual β diversity only.

**Table 2:** Results of best fit generalized least squares models relating β diversity and latitudinal turnover gradient (LTG) strength to mean annual precipitation from paleosols (Retallack, 2007), δ^18^O (‰) from benthic forams (mm/year; Zachos et al. 2001; 2008), length of North American Land Mammal Age subdivision, number of taxa sampled, sampling area (km^2^), and number of fossil localities.

Dependent Variable	Parameters of BestFit Model	Variance explainedby model (%)	t value	p
Beta Diversity	Mean annual precipitation (quadratic)	66.51	−3.25	0.005
	Length of NALMA subdivision		2.43	0.027
	Number of taxa		5.30	<0.001
Beta Diversity Residuals	Mean annual precipitation (quadratic)	26.48	−3.50	0.002
Beta Diversity Re-sampled	Mean annual precipitation (quadratic)	66.04	−2.39	0.029
	Length of NALMA subdivision		2.51	0.023
	Number of taxa		5.47	<0.001
Latitudinal Turnover Gradient Strength (LTGs)	Mean annual precipitation (quadratic)	46.76	−5.65	<0.001
	Area		−4.62	<0.001
	Number of taxa		−4.36	<0.001
	Area : Number of taxa		4.85	<0.001
LTG Residuals	Mean annual precipitation (linear)	37.48	−3.79	0.001
LTG Re-sampled	Number of taxa	28.59	−2.55	0.020

Mammalian latitudinal turnover gradients (LTGs) are weak prior to the late Miocene (Fig. 1D). Raw LTG strength (i.e. not detrended) peaks during late Miocene (Hemphillian) and late Pleistocene (Rancholabrean) (Fig. 1D). The best fit model includes mean annual precipitation (MAP) [66], number of taxa, area (km^2^) and an the interaction of area and the number of taxa, which explains 47% of the model variance (Table 2; Fig. 2C). LTG strength of late Cenozoic mammal species is statistically significantly explained by all four metrics (p<0.001; Table 2). Residual LTG strength is significantly explained only by MAP (p<0.05; Table 2; Fig. 2D). As above, re-sampling did not alleviate the effects of sampling bias on LTG strength (Table 2). In other words, even accounting for variables that describe potential sources of bias, a climatic variable (MAP) still explains a significant proportion of the variance.

**Figure 3:**
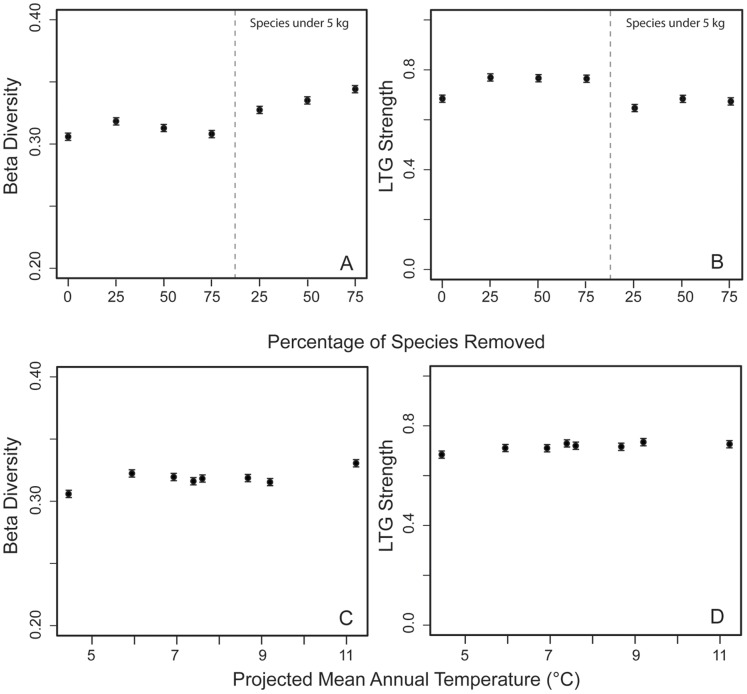
(A) β diversity (distance from centroid) and (B) latitudinal turnover gradients (LTG) strength of extant North American mammals under incomplete taxonomic sampling (removal of 25, 50, and 75% of species in sample) and body mass bias (removal of 25, 50, 75% of species smaller than 5 kg) and (C) β diversity (distance from centroid) and (D) latitudinal turnover gradients (LTG) strength of extant North American mammals under several International Panel on Climate Change scenarios (Special Reports on Emissions Scenarios).

β diversity is much lower for extant mammals than for extinct mammals (Fig. 3A). LTG strength for extant mammals is also greater than for early to mid Cenozoic fossil mammals, but similar to the values for the late Miocene and Pleistocene (Fig. 3B). Extant mammal β diversity shows a slight decrease under incomplete sampling and a slight increase under body-mass–bias sampling (Fig. 3A), but the change is much smaller than observed for fossil mammals. LTG strength does not appear to be significantly affected by the sample size reduction.

Our forecast models (which showed a strong fit to modern mammalian distributions, see Fig. S2A–C) show a slight increase in β diversity for extant mammals (Fig. 3C), but no substantial change in LTG strength compared to the present (Fig. 3D).

## Discussion

Spatiotemporal patterns of β diversity remain poorly studied despite being potentially very useful in conservation biology [17], [18], [70] and linkage to well-studied biogeographic phenomena such as latitudinal richness gradients [4]. Using an extensive analysis of past and present mammalian communities, we demonstrate that, over the past 36 Ma, spatiotemporal patterns of mammal community composition have varied by orders of magnitude in North America. Specifically, Cenozoic spatial turnover of mammal communities is explained by continental mean annual precipitation (MAP) (Fig. 2A–B), broadly supporting predictions drawn from published studies of modern terrestrial organisms [2], [70], [71] and our predictions outlined above.

Contemporary ecological theory predicts that mammal diversity either declines monotonically with productivity or shows a unimodal pattern, declining with both low and high productivity [1], [2], [70], [72]. Further, stronger latitudinal diversity gradients are associated with cooler, less productive environments [71] and steeper latitudinal climate gradients [1], [70]. Both sets of predictions assume that changes in climate, productivity, and seasonality influence rates of origination and extinction [72], [73], niche breadths [74], as well as the carrying capacity of the ecosystem [75], all factors that change the spatial turnover of terrestrial faunas [70]. Specifically, terrestrial organisms in low latitude, high productivity environments show low rates of speciation and extinction [73], high β diversity [2], [76], and weak or absent latitudinal diversity gradients [71]. In contrast, high latitude organisms show high rates of speciation and extinction [73], low β diversity [2], [76], and strong latitudinal diversity gradients [71]. Evolutionary history also plays a role in determining rates of spatial community turnover. Modern tropical organisms show faster turnover than their temperate counterparts regardless of the rate of environmental change [70]. Spatial and, by extension, temporal patterns of β diversity are the result of a mosaic of ecological and evolutionary processes.

Cenozoic fossil mammal β diversity peaked at intermediate values of mean annual precipitation and declined under both drier and wetter conditions (MAP; ∼450 mm per year; Fig. 2B), showing a similar shape to latitudinal diversity curves for modern mammals [71]. Mammal β diversity was similarly lowest during periods of relative cooling, including the early Oligocene and late Miocene, coincident with declining atmospheric CO_2_ [77]–[80] and, in the latter case, the expansion of ice sheets in the Northern Hemisphere [27], [36], strengthening of thermohaline circulation [27], [37], [81]–[84], and transition from C_3_ to C_4_ dominated ecosystems at middle latitudes [66], [85], [86]. Declining β diversity during the late Miocene is also coincident with increased maximum body mass [87], an ecologically relevant characteristic linked to lower ecosystem energy [88], [89]. Water is a key component in photosynthesis and therefore net primary productivity (NPP) and MAP are correlated at a global scale, showing an asymptotic relationship [90]. Our results therefore suggest that putatively lower energy ecosystems (e.g. early Oligocene, late Miocene) supported more spatially homogenous mammal faunas than putatively higher energy ecosystems (e.g. late Eocene, mid Miocene, mid Pliocene). Temporal changes in fossil mammal β diversity (this study) are therefore conceptually similar to spatial patterns observed in extant mammals.

Early Oligocene mammals had lower β diversity than expected based on MAP (Fig. 1C; Fig. 2A). The early Oligocene is associated with rapid global cooling [64] and expansion of open grassy ecosystems [91], which may have resulted in lower ecosystem energy. However, our taxonomic sample is the poorest for the early Oligocene; number of taxa is a significant predictor of fossil mammal β diversity (Table 2), suggesting some variation in preservation of species among NALMA subdivisions. Rarefied diversity also shows little change from the late Eocene to the early Oligocene [10]. However, our incomplete sampling trials show that removing even 75% of species reduces β diversity by a negligible amount (Fig. 3A), suggesting that at least some (but not all) of the observed decline in early Oligocene β diversity may have been climatically driven.

The magnitude of the latitudinal turnover gradient (LTG) for fossil mammals shows a temporally cyclic pattern that increases in amplitude during the late Cenozoic as well as a general trend toward stronger LTGs (Fig. 1D), coincident with the formation of ice on Svalbard at ∼15 Ma and perennial Arctic sea ice at ∼14 Ma, declining atmospheric CO_2_ [37], and declining terrestrial MAP (Fig. 2B). Specifically, LTGs are strongest when precipitation is lowest (putatively lower productivity environments) and weakest at when precipitation is highest (putatively high productivity environments; Fig. 2B), similar to modern mammals that show weak or absent latitudinal diversity gradients in the tropics and strong diversity gradients at mid to high latitudes [71]. Climate gradients are steeper at mid to high latitudes in North America due to the albedo of high latitude glaciation. Northern glaciation is an important means by which solar radiation is reflected from high latitudes, resulting in cool, low productivity Arctic environments [92], [93]. Mammal communities are sorted along a latitudinal axis according to their climatic tolerances and the process of abiotic filtering, whereby taxa meet the limits of their environmental tolerances and are excluded from communities farther north [94]. Although late Miocene sea and land ice thickness and extent were reduced compared to the modern, increasing northern albedo and strengthening of thermohaline circulation are coincident with that strengthening of mammal LTGs during the late Miocene (25–60% stronger than for any preceding NALMA; Fig. 1D) [27], [81]–[84].

At first glance, the Pliocene appears to be anomalous because the magnitude of the mammalian LTG declines dramatically (60–70% reduction in the magnitude of the LTG; Fig. 1D). However, evidence from fossil deposits on Ellesmere Island show that approximately 3.5 Ma the Pliocene Arctic was ∼14–22°C warmer than present [83], [95], [96] with an associated reduced volume of Arctic sea ice [27], [82]. Pliocene Arctic warming is similarly coincident with reduced richness gradients of marine zooplankton [81]. The Pliocene might therefore be the “exception” that proves the rule.

Under modern global warming, Arctic winter temperatures have increased at a greater rate than at southern latitudes [97]. Long-term projections suggest boosts in high latitude net primary productivity due to increasing nitrogen fertilization and increases in mean annual precipitation of 100–150 mm per year or 5–20% at middle to high latitudes [98]. From our analyses of fossil North American mammals and published studies of beta diversity [18], we therefore expect weakened climate gradients and thus weakened LTGs due to northward range shifting, and, in the long-term, declining β diversity under the influence of modern anthropogenic climate change. β diversity decline may be facilitated by the homogenization of communities due to any of the following (note the lack of mutual exclusivity): i) extinction of species with small geographic ranges and replacement with wide-ranging species, ii) evolution toward larger range sizes within species, and, iii) invasion by wide-ranging species even without the extinction of residents [18]. However, our climate space models that are based on SRES scenarios corresponding to absolute mean annual temperatures of 4.4–11.2°C (averaged across North and South America) did not show changes in mammal LTGs or β diversity (Fig. 3C–D). We suggest that climate space models (CSMs) are unlikely to accurately forecast the outcomes of anthropogenic climate change for modern mammals because current CSM algorithms do not incorporate microevolutionary, macroevolutionary, or ecological processes, such as niche shifts, niche creation, and differences in dispersal abilities that are inherent in the response of animals to climate change. However, even on modern ecological timescales, rapid evolutionary changes and niche shifts have been observed in native and invasive populations [41], and this local adaptation complicates the prediction of range shifts. On longer timescales, taxa adapt to new climates and the processes of speciation and extinction help form new terrestrial communities. Without the explicit inclusion of evolutionary parameters and historical data for the taxa of interest, we are unlikely to accurately predict long-term changes in terrestrial biodiversity patterns.

We have shown here that macroecological patterns of North American mammal community composition varied considerably over the past 35 million years in response to changes in global climate change and Arctic glaciation (Fig. 1C–D). Furthermore, our comparison of fossil evidence with climate-space forecast models (CSMs) suggests that CSMs (in which species are modeled to simply track climate variables) may distort the degree of community composition change we should expect in the future. A unifying ecological theory relating diversity to climate must address both the spatial and temporal dimensions of diversity, as well as both richness and community composition. However, studies of organismal richness are far more common than studies of community composition (β diversity), despite the importance of the latter in conservation and their vast potential for contributing to our understanding of the processes underlying modern biodiversity. Studying the community composition of fossil animals represents a new frontier in paleontological research with potential to truly inform modern conservation.

## Supporting Information

Figure S1 **Maps of North America showing the distribution of fossil localities for all sampled North American Land Mammal Age subdivisions.**
(TIF)

Figure S2 **Model fit statistics for climate space models of extant North American mammals.** Model performance was tested using area under the operating curve (A; AUC), the true skill statistics (B; TSS), and the proportion of correct classification (C).
(TIF)

Table S1 **Summary of Special Emissions Report Scenarios (SERs) to which we fit climate models for extant mammalian species.**
(DOCX)

Table S2 **List of mammalian taxa included and excluded from the species distribution models.**
(DOCX)

Appendix S1 **Sources for the majority of mammal occurrence data downloaded from the Fossilworks database.**
(DOCX)
