# Supplementary figures and images for: Mean Annual Precipitation Explains Spatiotemporal Patterns of Cenozoic Mammal Beta Diversity and Latitudinal Diversity Gradients in North America
Source: PLoS One. 2014 Sep 9;9(9):e106499. doi: 10.1371/journal.pone.0106499 (PMC4159275; doi:10.1371/journal.pone.0106499)

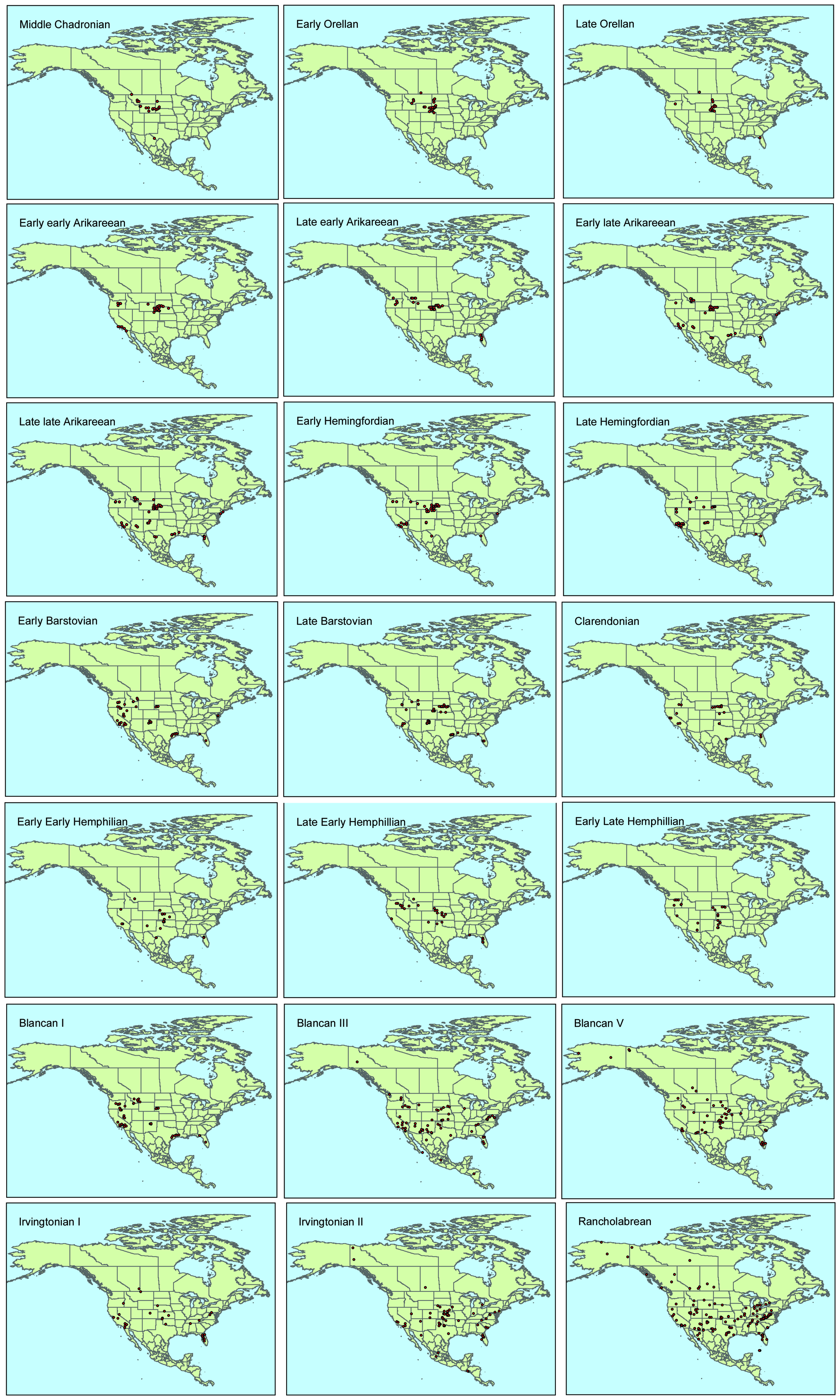

Supplement: Figure S1 — Maps of North America showing the distribution of fossil localities for all sampled North American Land Mammal Age subdivisions. (TIF) [file pone.0106499.s001.tif]

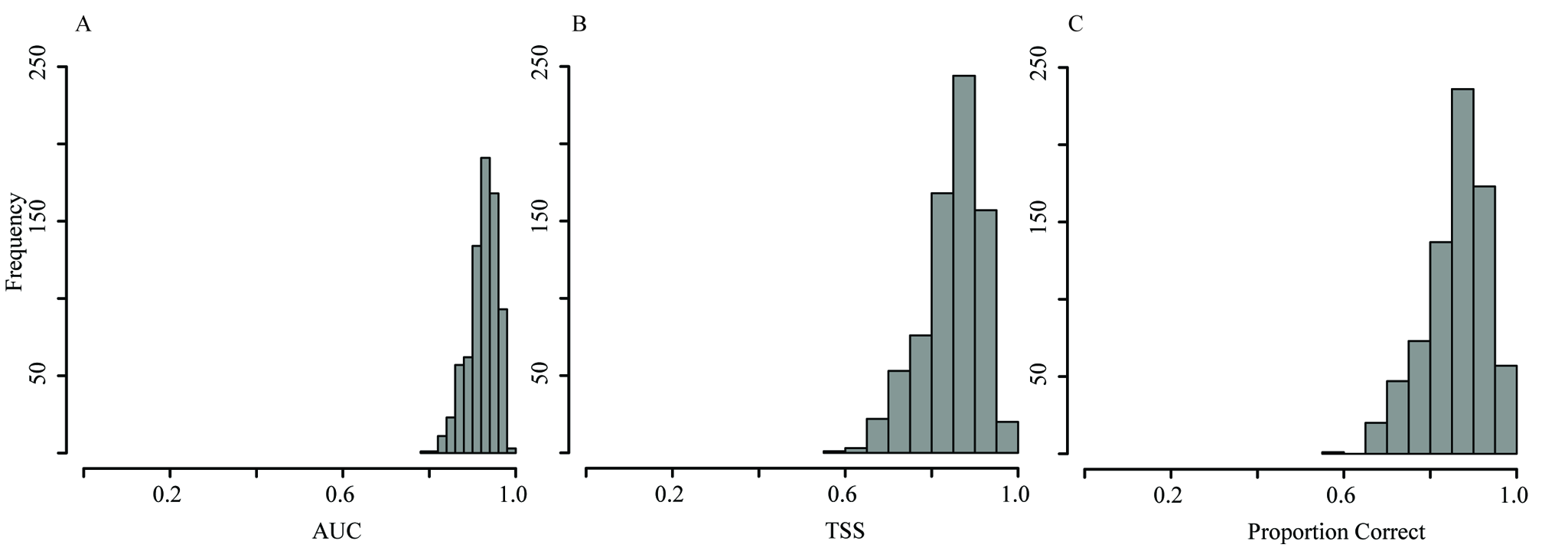

Supplement: Figure S2 — Model fit statistics for climate space models of extant North American mammals. Model performance was tested using area under the operating curve (A; AUC), the true skill statistics (B; TSS), and the proportion of correct classification (C). (TIF) [file pone.0106499.s002.tif]
