# Supplementary material for: Mean Annual Precipitation Explains Spatiotemporal Patterns of Cenozoic Mammal Beta Diversity and Latitudinal Diversity Gradients in North America
Source: PLoS One. 2014 Sep 9;9(9):e106499. doi: 10.1371/journal.pone.0106499 (PMC4159275; doi:10.1371/journal.pone.0106499)
Supplement: Table S1 — Summary of Special Emissions Report Scenarios (SERs) to which we fit climate models for extant mammalian species. (DOCX) [file pone.0106499.s003.docx]

| **Scenario** | **year** | **Mean Annual Temperature (˚C)** | **Standard Deviation** | **Standard Error** |
| --- | --- | --- | --- | --- |
|  | Present | 4.447 | 15.850 | 0.057 |
| B1_low | 2050s | 5.941 | 15.540 | 0.056 |
| B1 | 2050s | 6.926 | 15.420 | 0.056 |
| A1b | 2050s | 7.602 | 15.336 | 0.056 |
| A2 | 2050s | 8.674 | 15.163 | 0.055 |
| A1b | 2080s | 7.390 | 15.444 | 0.056 |
| A2 | 2080s | 9.196 | 15.198 | 0.055 |
| A2_top | 2080s | 11.225 | 14.721 | 0.053 |

**Table S1**. Summary of Special Emissions Report Scenarios (SERs) to which we fit climate models for extant mammalian species.
