# Supplementary material for: Mean Annual Precipitation Explains Spatiotemporal Patterns of Cenozoic Mammal Beta Diversity and Latitudinal Diversity Gradients in North America
Source: PLoS One. 2014 Sep 9;9(9):e106499. doi: 10.1371/journal.pone.0106499 (PMC4159275; doi:10.1371/journal.pone.0106499)
Supplement: Table S2 — List of mammalian taxa included and excluded from the species distribution models. (DOCX) [file pone.0106499.s004.docx]

**Table S2**. List of mammalian taxa included and excluded from the species distribution models.

|  |  |  |  |
| --- | --- | --- | --- |
| **Species** | **Status** |  |  |
| *Abrawayaomys ruschii* | Not found north of equator | | |
| *Abrothrix andinus* | Not found north of equator | | |
| *Abrocoma bennettii* | Not found north of equator | | |
| *Abrocoma boliviensis* | Not found north of equator | | |
| *Abrocoma cinerea* | Not found north of equator | | |
| *Abrothrix hershkovitzi* | Not found north of equator | | |
| *Abrothrix illuteus* | Not found north of equator | | |
| *Abrothrix jelskii* | Not found north of equator | | |
| *Abrothrix lanosus* | Not found north of equator | | |
| *Abrothrix longipilis* | Not found north of equator | | |
| *Abrothrix markhami* | Not found north of equator | | |
| *Abrothrix olivaceus* | Not found north of equator | | |
| *Abrothrix sanborni* | Not found north of equator | | |
| *Abrocoma schistacea* | Not found north of equator | | |
| *Aconaemys fuscus* | Not found north of equator | | |
| *Aconaemys porteri* | Not found north of equator | | |
| *Aconaemys sagei* | Not found north of equator | | |
| *Aepeomys lugens* | INCLUDED | |  |
| *Aepeomys reigi* | Less than 20 occurrences | | |
| *Akodon aerosus* | Not found north of equator | | |
| *Akodon affinis* | Less than 20 occurrences | | |
| *Akodon albiventer* | Not found north of equator | | |
| *Akodon aliquantulus* | Not found north of equator | | |
| *Akodon azarae* | Not found north of equator | | |
| *Akodon bogotensis* | INCLUDED | |  |
| *Akodon boliviensis* | Not found north of equator | | |
| *Akodon budini* | Not found north of equator | | |
| *Akodon cursor* | Not found north of equator | | |
| *Akodon dayi* | Not found north of equator | | |
| *Akodon dolores* | Not found north of equator | | |
| *Akodon fumeus* | Not found north of equator | | |
| *Akodon iniscatus* | Not found north of equator | | |
| *Akodon juninensis* | Not found north of equator | | |
| *Akodon kofordi* | Not found north of equator | | |
| *Akodon latebricola* | Not found north of equator | | |
| *Akodon lindberghi* | Not found north of equator | | |
| *Akodon lutescens* | Not found north of equator | | |
| *Akodon mimus* | Not found north of equator | | |
| *Akodon molinae* | Not found north of equator | | |
| *Akodon mollis* | Not found north of equator | | |
| *Akodon montensis* | Not found north of equator | | |
| *Akodon mystax* | Not found north of equator | | |
| *Akodon neocenus* | Not found north of equator | | |
| *Akodon oenos* | Not found north of equator | | |
| *Akodon orophilus* | Not found north of equator | | |
| *Akodon paranaensis* | Not found north of equator | | |
| *Akodon reigi* | Not found north of equator | | |
| *Akodon sanctipaulensis* | Not found north of equator | | |
| *Akodon serrensis* | Not found north of equator | | |
| *Akodon siberiae* | Not found north of equator | | |
| *Akodon simulator* | Not found north of equator | | |
| *Akodon spegazzinii* | Not found north of equator | | |
| *Akodon subfuscus* | Not found north of equator | | |
| *Akodon surdus* | Not found north of equator | | |
| *Akodon sylvanus* | Not found north of equator | | |
| *Akodon toba* | Not found north of equator | | |
| *Akodon torques* | Not found north of equator | | |
| *Akodon varius* | Not found north of equator | | |
| *Alces americanus* | INCLUDED | |  |
| *Alouatta belzebul* | Not found north of equator | | |
| *Alouatta caraya* | Not found north of equator | | |
| *Alouatta coibensis* | Less than 20 occurrences | | |
| *Alouatta guariba* | Not found north of equator | | |
| *Alouatta nigerrima* | Not found north of equator | | |
| *Alouatta palliata* | INCLUDED | |  |
| *Alouatta pigra* | INCLUDED | |  |
| *Alouatta sara* | Not found north of equator | | |
| *Alouatta seniculus* | INCLUDED | |  |
| *Ametrida centurio* | INCLUDED | |  |
| *Ammospermophilus harrisii* | INCLUDED | |  |
| *Ammospermophilus insularis* | Less than 20 occurrences | | |
| *Ammospermophilus interpres* | INCLUDED | |  |
| *Ammospermophilus leucurus* | INCLUDED | |  |
| *Ammospermophilus nelsoni* | Less than 20 occurrences | | |
| *Amorphochilus schnablii* | Not found north of equator | | |
| *Amphinectomys savamis* | Not found north of equator | | |
| *Andalgalomys olrogi* | Not found north of equator | | |
| *Andalgalomys pearsoni* | Not found north of equator | | |
| *Andalgalomys roigi* | Not found north of equator | | |
| *Andinomys edax* | Not found north of equator | | |
| *Anotomys leander* | Not found north of equator | | |
| *Anoura caudifer* | INCLUDED | |  |
| *Anoura cultrata* | INCLUDED | |  |
| *Anoura geoffroyi* | INCLUDED | |  |
| *Anoura latidens* | INCLUDED | |  |
| *Anoura luismanueli* | Less than 20 occurrences | | |
| *Antilocapra americana* | INCLUDED | |  |
| *Antrozous pallidus* | INCLUDED | |  |
| *Aotus azarae* | Not found north of equator | | |
| *Aotus lemurinus* | INCLUDED | |  |
| *Aotus miconax* | Not found north of equator | | |
| *Aotus nancymaae* | Not found north of equator | | |
| *Aotus nigriceps* | Not found north of equator | | |
| *Aotus trivirgatus* | INCLUDED | |  |
| *Aotus vociferans* | INCLUDED | |  |
| *Aplodontia rufa* | INCLUDED | |  |
| *Arborimus albipes* | Less than 20 occurrences | | |
| *Arborimus longicaudus* | Less than 20 occurrences | | |
| *Arborimus pomo* | Less than 20 occurrences | | |
| *Ardops nichollsi* | Less than 20 occurrences | | |
| *Ariteus flavescens* | Less than 20 occurrences | | |
| *Artibeus amplus* | INCLUDED | |  |
| *Artibeus anderseni* | Not found north of equator | | |
| *Artibeus aztecus* | INCLUDED | |  |
| *Artibeus cinereus* | INCLUDED | |  |
| *Artibeus concolor* | INCLUDED | |  |
| *Artibeus fimbriatus* | Not found north of equator | | |
| *Artibeus fraterculus* | Not found north of equator | | |
| *Artibeus glaucus* | INCLUDED | |  |
| *Artibeus gnomus* | INCLUDED | |  |
| *Artibeus hirsutus* | INCLUDED | |  |
| *Artibeus inopinatus* | Less than 20 occurrences | | |
| *Artibeus jamaicensis* | INCLUDED | |  |
| *Artibeus lituratus* | INCLUDED | |  |
| *Artibeus obscurus* | INCLUDED | |  |
| *Artibeus phaeotis* | INCLUDED | |  |
| *Artibeus toltecus* | INCLUDED | |  |
| *Artibeus triomylus* | INCLUDED | |  |
| *Artibeus watsoni* | INCLUDED | |  |
| *Ateles belzebuth* | INCLUDED | |  |
| *Ateles chamek* | Not found north of equator | | |
| *Ateles fusciceps* | INCLUDED | |  |
| *Ateles geoffroyi* | INCLUDED | |  |
| *Ateles hybridus* | INCLUDED | |  |
| *Ateles marginatus* | Not found north of equator | | |
| *Atelocynus microtis* | INCLUDED | |  |
| *Ateles paniscus* | INCLUDED | |  |
| *Auliscomys boliviensis* | Not found north of equator | | |
| *Auliscomys pictus* | Not found north of equator | | |
| *Auliscomys sublimis* | Not found north of equator | | |
| *Baiomys musculus* | INCLUDED | |  |
| *Baiomys taylori* | INCLUDED | |  |
| *Balantiopteryx infusca* | Less than 20 occurrences | | |
| *Balantiopteryx io* | INCLUDED | |  |
| *Balantiopteryx plicata* | INCLUDED | |  |
| *Bassaricyon alleni* | Not found north of equator | | |
| *Bassariscus astutus* | INCLUDED | |  |
| *Bassaricyon beddardi* | INCLUDED | |  |
| *Bassaricyon gabbii* | INCLUDED | |  |
| *Bassaricyon lasius* | Less than 20 occurrences | | |
| *Bassaricyon pauli* | Less than 20 occurrences | | |
| *Bassariscus sumichrasti* | INCLUDED | |  |
| *Bauerus dubiaquercus* | INCLUDED | |  |
| *Bibimys chacoensis* | Not found north of equator | | |
| *Bibimys labiosus* | Not found north of equator | | |
| *Bibimys torresi* | Not found north of equator | | |
| *Blarinomys breviceps* | Not found north of equator | | |
| *Blarina carolinensis* | INCLUDED | |  |
| *Blarina hylophaga* | INCLUDED | |  |
| *Blastocerus dichotomus* | Not found north of equator | | |
| *Bos bison* | INCLUDED | |  |
| *Brachyteles arachnoides* | Not found north of equator | | |
| *Brachyphylla cavernarum* | Less than 20 occurrences | | |
| *Brachyteles hypoxanthus* | Not found north of equator | | |
| *Brachylagus idahoensis* | INCLUDED | |  |
| *Brachyphylla nana* | INCLUDED | |  |
| *Bradypus torquatus* | Not found north of equator | | |
| *Bradypus tridactylus* | INCLUDED | |  |
| *Bradypus variegatus* | INCLUDED | |  |
| *Brucepattersonius albinasus* | Not found north of equator | | |
| *Brucepattersonius griserufescens* | Not found north of equator | | |
| *Brucepattersonius guarani* | Not found north of equator | | |
| *Brucepattersonius igniventris* | Not found north of equator | | |
| *Brucepattersonius iheringi* | Not found north of equator | | |
| *Brucepattersonius misionensis* | Not found north of equator | | |
| *Brucepattersonius paradisus* | Not found north of equator | | |
| *Brucepattersonius soricinus* | Not found north of equator | | |
| *Cabassous centralis* | INCLUDED | |  |
| *Cabassous chacoensis* | Not found north of equator | | |
| *Cabassous tatouay* | Not found north of equator | | |
| *Cabassous unicinctus* | INCLUDED | |  |
| *Cacajao calvus* | Not found north of equator | | |
| *Cacajao melanocephalus* | Less than 20 occurrences | | |
| *Caenolestes caniventer* | Not found north of equator | | |
| *Caenolestes convelatus* | Less than 20 occurrences | | |
| *Caenolestes fuliginosus* | INCLUDED | |  |
| *Callithrix acariensis* | Not found north of equator | | |
| *Callithrix argentata* | Not found north of equator | | |
| *Callicebus aureipalatii* | Not found north of equator | | |
| *Callithrix aurita* | Not found north of equator | | |
| *Callicebus baptista* | Not found north of equator | | |
| *Callicebus barbarabrownae* | Not found north of equator | | |
| *Callicebus bernhardi* | Not found north of equator | | |
| *Callicebus brunneus* | Not found north of equator | | |
| *Callicebus caligatus* | Not found north of equator | | |
| *Callithrix chrysoleuca* | Not found north of equator | | |
| *Callicebus cinerascens* | Not found north of equator | | |
| *Callicebus coimbrai* | Not found north of equator | | |
| *Callicebus cupreus* | Not found north of equator | | |
| *Callicebus discolor* | Not found north of equator | | |
| *Callicebus donacophilus* | Not found north of equator | | |
| *Callicebus dubius* | Not found north of equator | | |
| *Callithrix emiliae* | Not found north of equator | | |
| *Callithrix flaviceps* | Not found north of equator | | |
| *Callithrix geoffroyi* | Not found north of equator | | |
| *Callimico goeldii* | Not found north of equator | | |
| *Callicebus hoffmannsi* | Not found north of equator | | |
| *Callithrix humeralifera* | Not found north of equator | | |
| *Callibella humilis* | Not found north of equator | | |
| *Callithrix intermedia* | Not found north of equator | | |
| *Callithrix jacchus* | Not found north of equator | | |
| *Callithrix kuhlii* | Not found north of equator | | |
| *Callithrix leucippe* | Not found north of equator | | |
| *Callicebus lucifer* | Not found north of equator | | |
| *Callicebus lugens* | INCLUDED | |  |
| *Callithrix manicorensis* | Not found north of equator | | |
| *Callithrix marcai* | Not found north of equator | | |
| *Callithrix mauesi* | Not found north of equator | | |
| *Callicebus medemi* | Not found north of equator | | |
| *Callithrix melanura* | Not found north of equator | | |
| *Callicebus modestus* | Not found north of equator | | |
| *Callicebus moloch* | Not found north of equator | | |
| *Callithrix nigriceps* | Not found north of equator | | |
| *Callicebus oenanthe* | Not found north of equator | | |
| *Callicebus olallae* | Not found north of equator | | |
| *Callicebus ornatus* | Less than 20 occurrences | | |
| *Callicebus pallescens* | Not found north of equator | | |
| *Callithrix penicillata* | Not found north of equator | | |
| *Callicebus personatus* | Not found north of equator | | |
| *Callistomys pictus* | Not found north of equator | | |
| *Callicebus purinus* | Not found north of equator | | |
| *Callithrix pygmaea* | Not found north of equator | | |
| *Callicebus regulus* | Not found north of equator | | |
| *Callithrix saterei* | Not found north of equator | | |
| *Callicebus stephennashi* | Not found north of equator | | |
| *Callicebus torquatus* | Not found north of equator | | |
| *Calomys boliviae* | Not found north of equator | | |
| *Calomys callidus* | Not found north of equator | | |
| *Calomys callosus* | Not found north of equator | | |
| *Calomys hummelincki* | INCLUDED | |  |
| *Calomys laucha* | Less than 20 occurrences | | |
| *Calomys lepidus* | Not found north of equator | | |
| *Calomys musculinus* | Not found north of equator | | |
| *Calomys sorellus* | Not found north of equator | | |
| *Calomys tener* | Not found north of equator | | |
| *Calomys tocantinsi* | Not found north of equator | | |
| *Caluromys derbianus* | INCLUDED | |  |
| *Caluromysiops irrupta* | Not found north of equator | | |
| *Caluromys lanatus* | INCLUDED | |  |
| *Caluromys philander* | INCLUDED | |  |
| *Calyptophractus retusus* | Not found north of equator | | |
| *Canis latrans* | INCLUDED | |  |
| *Canis lupus* | INCLUDED | |  |
| *Canis rufus* | INCLUDED | |  |
| *Capromys pilorides* | Less than 20 occurrences | | |
| *Carollia brevicauda* | INCLUDED | |  |
| *Carollia castanea* | INCLUDED | |  |
| *Carollia manu* | Not found north of equator | | |
| *Carollia perspicillata* | INCLUDED | |  |
| *Carollia sowelli* | INCLUDED | |  |
| *Carollia subrufa* | INCLUDED | |  |
| *Carterodon sulcidens* | Not found north of equator | | |
| *Castor canadensis* | INCLUDED | |  |
| *Catagonus wagneri* | Not found north of equator | | |
| *Cavia aperea* | INCLUDED | |  |
| *Cavia fulgida* | Not found north of equator | | |
| *Cavia magna* | Not found north of equator | | |
| *Cavia porcellus* | INCLUDED | |  |
| *Cavia tschudii* | Not found north of equator | | |
| *Cebus albifrons* | INCLUDED | |  |
| *Cebus apella* | INCLUDED | |  |
| *Cebus capucinus* | INCLUDED | |  |
| *Cebus kaapori* | Not found north of equator | | |
| *Cebus libidinosus* | Not found north of equator | | |
| *Cebus nigritus* | Not found north of equator | | |
| *Cebus olivaceus* | INCLUDED | |  |
| *Cebus xanthosternos* | Not found north of equator | | |
| *Centronycteris centralis* | INCLUDED | |  |
| *Centronycteris maximiliani* | INCLUDED | |  |
| *Centurio senex* | INCLUDED | |  |
| *Cerdocyon thous* | INCLUDED | |  |
| *Cervus canadensis* | INCLUDED | |  |
| *Chaetodipus arenarius* | INCLUDED | |  |
| *Chaetodipus artus* | Less than 20 occurrences | | |
| *Chaetodipus baileyi* | INCLUDED | |  |
| *Chaetodipus californicus* | INCLUDED | |  |
| *Chaetodipus eremicus* | INCLUDED | |  |
| *Chaetodipus fallax* | Less than 20 occurrences | | |
| *Chaetodipus formosus* | INCLUDED | |  |
| *Chaetodipus goldmani* | Less than 20 occurrences | | |
| *Chaetodipus hispidus* | INCLUDED | |  |
| *Chaetodipus intermedius* | INCLUDED | |  |
| *Chaetodipus lineatus* | Less than 20 occurrences | | |
| *Chaetophractus nationi* | Not found north of equator | | |
| *Chaetodipus nelsoni* | INCLUDED | |  |
| *Chaetodipus penicillatus* | INCLUDED | |  |
| *Chaetodipus pernix* | INCLUDED | |  |
| *Chaetodipus rudinoris* | INCLUDED | |  |
| *Chaetodipus spinatus* | INCLUDED | |  |
| *Chaetomys subspinosus* | Not found north of equator | | |
| *Chaetophractus vellerosus* | Not found north of equator | | |
| *Chaetophractus villosus* | Not found north of equator | | |
| *Chelemys delfini* | Not found north of equator | | |
| *Chelemys macronyx* | Not found north of equator | | |
| *Chelemys megalonyx* | Not found north of equator | | |
| *Chibchanomys orcesi* | Not found north of equator | | |
| *Chibchanomys trichotis* | INCLUDED | |  |
| *Chilomys instans* | INCLUDED | |  |
| *Chilonatalus micropus* | INCLUDED | |  |
| *Chilonatalus tumidifrons* | INCLUDED | |  |
| *Chinchilla chinchilla* | Not found north of equator | | |
| *Chinchilla lanigera* | Not found north of equator | | |
| *Chinchillula sahamae* | Not found north of equator | | |
| *Chiropotes albinasus* | Not found north of equator | | |
| *Chiropotes chiropotes* | INCLUDED | |  |
| *Chiroderma doriae* | Not found north of equator | | |
| *Chiroderma improvisum* | Less than 20 occurrences | | |
| *Chironectes minimus* | INCLUDED | |  |
| *Chiroderma salvini* | INCLUDED | |  |
| *Chiropotes satanas* | Not found north of equator | | |
| *Chiroderma trinitatum* | INCLUDED | |  |
| *Chiropotes utahickae* | Not found north of equator | | |
| *Chiroderma villosum* | INCLUDED | |  |
| *Chlamyphorus truncatus* | Not found north of equator | | |
| *Choeroniscus godmani* | INCLUDED | |  |
| *Choeronycteris mexicana* | INCLUDED | |  |
| *Choeroniscus minor* | INCLUDED | |  |
| *Choeroniscus periosus* | Less than 20 occurrences | | |
| *Choloepus didactylus* | INCLUDED | |  |
| *Choloepus hoffmanni* | INCLUDED | |  |
| *Chrotopterus auritus* | INCLUDED | |  |
| *Chrysocyon brachyurus* | Not found north of equator | | |
| *Clyomys bishopi* | Not found north of equator | | |
| *Clyomys laticeps* | Not found north of equator | | |
| *Coendou bicolor* | Less than 20 occurrences | | |
| *Coendou nycthemera* | Not found north of equator | | |
| *Coendou prehensilis* | INCLUDED | |  |
| *Coendou rothschildi* | Less than 20 occurrences | | |
| *Condylura cristata* | INCLUDED | |  |
| *Conepatus chinga* | Not found north of equator | | |
| *Conepatus humboldtii* | Not found north of equator | | |
| *Conepatus leuconotus* | INCLUDED | |  |
| *Conepatus semistriatus* | INCLUDED | |  |
| *Cormura brevirostris* | INCLUDED | |  |
| *Corynorhinus mexicanus* | INCLUDED | |  |
| *Corynorhinus rafinesquii* | INCLUDED | |  |
| *Corynorhinus townsendii* | INCLUDED | |  |
| *Cratogeomys castanops* | INCLUDED | |  |
| *Cratogeomys fumosus* | Less than 20 occurrences | | |
| *Cratogeomys gymnurus* | Less than 20 occurrences | | |
| *Cratogeomys merriami* | Less than 20 occurrences | | |
| *Cratogeomys neglectus* | Less than 20 occurrences | | |
| *Cratogeomys tylorhinus* | Less than 20 occurrences | | |
| *Cratogeomys zinseri* | Less than 20 occurrences | | |
| *Cryptotis alticola* | Less than 20 occurrences | | |
| *Cryptotis colombiana* | Less than 20 occurrences | | |
| *Cryptotis endersi* | Less than 20 occurrences | | |
| *Cryptotis equatoris* | Not found north of equator | | |
| *Cryptotis goldmani* | Less than 20 occurrences | | |
| *Cryptotis goodwini* | Less than 20 occurrences | | |
| *Cryptotis gracilis* | Less than 20 occurrences | | |
| *Cryptotis griseoventris* | Less than 20 occurrences | | |
| *Cryptotis hondurensis* | Less than 20 occurrences | | |
| *Cryptotis magna* | Less than 20 occurrences | | |
| *Cryptotis mayensis* | INCLUDED | |  |
| *Cryptotis medellinia* | Less than 20 occurrences | | |
| *Cryptotis mera* | Less than 20 occurrences | | |
| *Cryptotis meridensis* | Less than 20 occurrences | | |
| *Cryptotis merriami* | INCLUDED | |  |
| *Cryptotis mexicana* | Less than 20 occurrences | | |
| *Cryptotis montivaga* | Not found north of equator | | |
| *Cryptotis nelsoni* | Less than 20 occurrences | | |
| *Cryptotis nigrescens* | INCLUDED | |  |
| *Cryptotis obscura* | Less than 20 occurrences | | |
| *Cryptotis parva* | INCLUDED | |  |
| *Cryptotis peregrina* | Less than 20 occurrences | | |
| *Cryptotis phillipsii* | Less than 20 occurrences | | |
| *Cryptotis squamipes* | Less than 20 occurrences | | |
| *Cryptotis tamensis* | Less than 20 occurrences | | |
| *Cryptotis thomasi* | Less than 20 occurrences | | |
| *Ctenomys argentinus* | Not found north of equator | | |
| *Ctenomys australis* | Not found north of equator | | |
| *Ctenomys azarae* | Not found north of equator | | |
| *Ctenomys bergi* | Not found north of equator | | |
| *Ctenomys boliviensis* | Not found north of equator | | |
| *Ctenomys bonettoi* | Not found north of equator | | |
| *Ctenomys brasiliensis* | Not found north of equator | | |
| *Ctenomys colburni* | Not found north of equator | | |
| *Ctenomys conoveri* | Not found north of equator | | |
| *Ctenomys dorbignyi* | Not found north of equator | | |
| *Ctenomys dorsalis* | Not found north of equator | | |
| *Ctenomys emilianus* | Not found north of equator | | |
| *Ctenomys flamarioni* | Not found north of equator | | |
| *Ctenomys frater* | Not found north of equator | | |
| *Ctenomys fulvus* | Not found north of equator | | |
| *Ctenomys goodfellowi* | Not found north of equator | | |
| *Ctenomys haigi* | Not found north of equator | | |
| *Ctenomys knighti* | Not found north of equator | | |
| *Ctenomys latro* | Not found north of equator | | |
| *Ctenomys leucodon* | Not found north of equator | | |
| *Ctenomys lewisi* | Not found north of equator | | |
| *Ctenomys magellanicus* | Not found north of equator | | |
| *Ctenomys maulinus* | Not found north of equator | | |
| *Ctenomys mendocinus* | Not found north of equator | | |
| *Ctenomys minutus* | Not found north of equator | | |
| *Ctenomys occultus* | Not found north of equator | | |
| *Ctenomys opimus* | Not found north of equator | | |
| *Ctenomys osvaldoreigi* | Not found north of equator | | |
| *Ctenomys pearsoni* | Not found north of equator | | |
| *Ctenomys perrensi* | Not found north of equator | | |
| *Ctenomys peruanus* | Not found north of equator | | |
| *Ctenomys pontifex* | Not found north of equator | | |
| *Ctenomys porteousi* | Not found north of equator | | |
| *Ctenomys rionegrensis* | Not found north of equator | | |
| *Ctenomys saltarius* | Not found north of equator | | |
| *Ctenomys sericeus* | Not found north of equator | | |
| *Ctenomys sociabilis* | Not found north of equator | | |
| *Ctenomys steinbachi* | Not found north of equator | | |
| *Ctenomys talarum* | Not found north of equator | | |
| *Ctenomys torquatus* | Not found north of equator | | |
| *Ctenomys tuconax* | Not found north of equator | | |
| *Ctenomys tucumanus* | Not found north of equator | | |
| *Ctenomys validus* | Not found north of equator | | |
| *Cuniculus paca* | INCLUDED | |  |
| *Cuniculus taczanowskii* | INCLUDED | |  |
| *Cuscomys ashaninka* | Not found north of equator | | |
| *Cyclopes didactylus* | INCLUDED | |  |
| *Cynomops abrasus* | INCLUDED | |  |
| *Cynomys gunnisoni* | INCLUDED | |  |
| *Cynomys leucurus* | INCLUDED | |  |
| *Cynomys ludovicianus* | INCLUDED | |  |
| *Cynomops mexicanus* | Less than 20 occurrences | | |
| *Cynomops paranus* | INCLUDED | |  |
| *Cynomys parvidens* | Less than 20 occurrences | | |
| *Cynomops planirostris* | INCLUDED | |  |
| *Cyttarops alecto* | INCLUDED | |  |
| *Dactylomys boliviensis* | Not found north of equator | | |
| *Dactylomys dactylinus* | Less than 20 occurrences | | |
| *Dactylomys peruanus* | Not found north of equator | | |
| *Dasyprocta azarae* | Not found north of equator | | |
| *Dasyprocta coibae* | Less than 20 occurrences | | |
| *Dasyprocta fuliginosa* | INCLUDED | |  |
| *Dasyprocta guamara* | Less than 20 occurrences | | |
| *Dasypus hybridus* | Not found north of equator | | |
| *Dasyprocta kalinowskii* | Not found north of equator | | |
| *Dasypus kappleri* | INCLUDED | |  |
| *Dasyprocta leporina* | INCLUDED | |  |
| *Dasyprocta mexicana* | INCLUDED | |  |
| *Dasypus novemcinctus* | INCLUDED | |  |
| *Dasypus pilosus* | Not found north of equator | | |
| *Dasyprocta prymnolopha* | Not found north of equator | | |
| *Dasyprocta punctata* | INCLUDED | |  |
| *Dasyprocta ruatanica* | Not found north of equator | | |
| *Dasypus sabanicola* | INCLUDED | |  |
| *Dasypus septemcinctus* | Not found north of equator | | |
| *Delomys dorsalis* | Not found north of equator | | |
| *Delomys sublineatus* | Not found north of equator | | |
| *Deltamys kempi* | Not found north of equator | | |
| *Desmodus rotundus* | INCLUDED | |  |
| *Diaemus youngi* | INCLUDED | |  |
| *Diclidurus albus* | INCLUDED | |  |
| *Diclidurus ingens* | INCLUDED | |  |
| *Diclidurus isabellus* | INCLUDED | |  |
| *Diclidurus scutatus* | INCLUDED | |  |
| *Dicrostonyx groenlandicus* | INCLUDED | |  |
| *Dicrostonyx hudsonius* | INCLUDED | |  |
| *Dicrostonyx nelsoni* | INCLUDED | |  |
| *Dicrostonyx nunatakensis* | INCLUDED | |  |
| *Dicrostonyx richardsoni* | INCLUDED | |  |
| *Dicrostonyx unalascensis* | Less than 20 occurrences | | |
| *Didelphis albiventris* | INCLUDED | |  |
| *Didelphis aurita* | Not found north of equator | | |
| *Didelphis imperfecta* | INCLUDED | |  |
| *Didelphis marsupialis* | INCLUDED | |  |
| *Didelphis pernigra* | INCLUDED | |  |
| *Didelphis virginiana* | INCLUDED | |  |
| *Dinomys branickii* | INCLUDED | |  |
| *Diphylla ecaudata* | INCLUDED | |  |
| *Diplomys caniceps* | Less than 20 occurrences | | |
| *Diplomys labilis* | Less than 20 occurrences | | |
| *Diplomys rufodorsalis* | Less than 20 occurrences | | |
| *Dipodomys agilis* | Less than 20 occurrences | | |
| *Dipodomys californicus* | INCLUDED | |  |
| *Dipodomys compactus* | Less than 20 occurrences | | |
| *Dipodomys deserti* | INCLUDED | |  |
| *Dipodomys elator* | Less than 20 occurrences | | |
| *Dipodomys gravipes* | Less than 20 occurrences | | |
| *Dipodomys heermanni* | Less than 20 occurrences | | |
| *Dipodomys ingens* | Less than 20 occurrences | | |
| *Dipodomys merriami* | INCLUDED | |  |
| *Dipodomys microps* | INCLUDED | |  |
| *Dipodomys nelsoni* | INCLUDED | |  |
| *Dipodomys nitratoides* | Less than 20 occurrences | | |
| *Dipodomys ordii* | INCLUDED | |  |
| *Dipodomys panamintinus* | Less than 20 occurrences | | |
| *Dipodomys phillipsii* | INCLUDED | |  |
| *Dipodomys simulans* | INCLUDED | |  |
| *Dipodomys spectabilis* | INCLUDED | |  |
| *Dipodomys stephensi* | Less than 20 occurrences | | |
| *Dipodomys venustus* | Less than 20 occurrences | | |
| *Dolichotis patagonum* | Not found north of equator | | |
| *Dolichotis salinicola* | Not found north of equator | | |
| *Dromiciops gliroides* | Not found north of equator | | |
| *Echimys chrysurus* | INCLUDED | |  |
| *Echinoprocta rufescens* | Less than 20 occurrences | | |
| *Echimys saturnus* | Not found north of equator | | |
| *Echimys semivillosus* | INCLUDED | |  |
| *Ectophylla alba* | INCLUDED | |  |
| *Eira barbara* | INCLUDED | |  |
| *Eligmodontia moreni* | Not found north of equator | | |
| *Eligmodontia morgani* | Not found north of equator | | |
| *Eligmodontia puerulus* | Not found north of equator | | |
| *Eligmodontia typus* | Not found north of equator | | |
| *Enchisthenes hartii* | INCLUDED | |  |
| *Eptesicus andinus* | INCLUDED | |  |
| *Eptesicus brasiliensis* | INCLUDED | |  |
| *Eptesicus chiriquinus* | INCLUDED | |  |
| *Eptesicus diminutus* | Not found north of equator | | |
| *Eptesicus furinalis* | INCLUDED | |  |
| *Eptesicus fuscus* | INCLUDED | |  |
| *Eptesicus guadeloupensis* | Less than 20 occurrences | | |
| *Eptesicus innoxius* | Not found north of equator | | |
| *Erethizon dorsatum* | INCLUDED | |  |
| *Erophylla bombifrons* | Less than 20 occurrences | | |
| *Erophylla sezekorni* | Less than 20 occurrences | | |
| *Euderma maculatum* | INCLUDED | |  |
| *Eumops auripendulus* | INCLUDED | |  |
| *Eumops bonariensis* | INCLUDED | |  |
| *Eumops dabbenei* | INCLUDED | |  |
| *Eumops glaucinus* | INCLUDED | |  |
| *Eumops hansae* | INCLUDED | |  |
| *Eumops maurus* | INCLUDED | |  |
| *Eumops perotis* | INCLUDED | |  |
| *Eumops trumbulli* | INCLUDED | |  |
| *Eumops underwoodi* | INCLUDED | |  |
| *Euneomys chinchilloides* | Not found north of equator | | |
| *Euneomys fossor* | Not found north of equator | | |
| *Euneomys mordax* | Not found north of equator | | |
| *Euneomys petersoni* | Not found north of equator | | |
| *Euphractus sexcinctus* | Less than 20 occurrences | | |
| *Euryzygomatomys spinosus* | Not found north of equator | | |
| *Furipterus horrens* | INCLUDED | |  |
| *Galea flavidens* | Not found north of equator | | |
| *Galenomys garleppi* | Not found north of equator | | |
| *Galea musteloides* | Not found north of equator | | |
| *Galea spixii* | Not found north of equator | | |
| *Galictis cuja* | Not found north of equator | | |
| *Galictis vittata* | INCLUDED | |  |
| *Geocapromys brownii* | Less than 20 occurrences | | |
| *Geocapromys ingrahami* | Less than 20 occurrences | | |
| *Geomys arenarius* | INCLUDED | |  |
| *Geomys attwateri* | Less than 20 occurrences | | |
| *Geomys breviceps* | INCLUDED | |  |
| *Geomys bursarius* | INCLUDED | |  |
| *Geomys knoxjonesi* | Less than 20 occurrences | | |
| *Geomys personatus* | INCLUDED | |  |
| *Geomys pinetis* | INCLUDED | |  |
| *Geomys streckeri* | Less than 20 occurrences | | |
| *Geomys texensis* | Less than 20 occurrences | | |
| *Geoxus valdivianus* | Not found north of equator | | |
| *Glaucomys sabrinus* | INCLUDED | |  |
| *Glaucomys volans* | INCLUDED | |  |
| *Glironia venusta* | Not found north of equator | | |
| *Glossophaga commissarisi* | INCLUDED | |  |
| *Glossophaga leachii* | INCLUDED | |  |
| *Glossophaga longirostris* | INCLUDED | |  |
| *Glossophaga morenoi* | INCLUDED | |  |
| *Glossophaga soricina* | INCLUDED | |  |
| *Glyphonycteris behnii* | Not found north of equator | | |
| *Glyphonycteris daviesi* | INCLUDED | |  |
| *Glyphonycteris sylvestris* | INCLUDED | |  |
| *Gracilinanus aceramarcae* | Not found north of equator | | |
| *Gracilinanus agilis* | Less than 20 occurrences | | |
| *Gracilinanus dryas* | Less than 20 occurrences | | |
| *Gracilinanus emiliae* | Less than 20 occurrences | | |
| *Gracilinanus marica* | INCLUDED | |  |
| *Gracilinanus microtarsus* | Not found north of equator | | |
| *Graomys domorum* | Not found north of equator | | |
| *Graomys edithae* | Not found north of equator | | |
| *Graomys griseoflavus* | Not found north of equator | | |
| *Gulo gulo* | INCLUDED | |  |
| *Habromys chinanteco* | Less than 20 occurrences | | |
| *Habromys lepturus* | Less than 20 occurrences | | |
| *Habromys lophurus* | Less than 20 occurrences | | |
| *Habromys simulatus* | Less than 20 occurrences | | |
| *Handleyomys fuscatus* | Less than 20 occurrences | | |
| *Handleyomys intectus* | Less than 20 occurrences | | |
| *Herpestes javanicus* | INCLUDED | |  |
| *Heteromys anomalus* | INCLUDED | |  |
| *Heteromys australis* | INCLUDED | |  |
| *Heteromys desmarestianus* | INCLUDED | |  |
| *Heteromys gaumeri* | INCLUDED | |  |
| *Heteromys nelsoni* | Less than 20 occurrences | | |
| *Heteromys oasicus* | Less than 20 occurrences | | |
| *Heteromys oresterus* | Less than 20 occurrences | | |
| *Heteromys teleus* | Not found north of equator | | |
| *Hippocamelus antisensis* | Not found north of equator | | |
| *Hippocamelus bisulcus* | Not found north of equator | | |
| *Histiotus alienus* | Not found north of equator | | |
| *Histiotus humboldti* | INCLUDED | |  |
| *Histiotus macrotus* | Not found north of equator | | |
| *Histiotus montanus* | INCLUDED | |  |
| *Histiotus velatus* | Not found north of equator | | |
| *Hodomys alleni* | INCLUDED | |  |
| *Holochilus brasiliensis* | Not found north of equator | | |
| *Holochilus chacarius* | Not found north of equator | | |
| *Holochilus sciureus* | INCLUDED | |  |
| *Hoplomys gymnurus* | Less than 20 occurrences | | |
| *Hydrochoerus hydrochaeris* | INCLUDED | |  |
| *Hyladelphys kalinowskii* | Less than 20 occurrences | | |
| *Hylonycteris underwoodi* | INCLUDED | |  |
| *Ichthyomys hydrobates* | INCLUDED | |  |
| *Ichthyomys pittieri* | Less than 20 occurrences | | |
| *Ichthyomys stolzmanni* | Not found north of equator | | |
| *Ichthyomys tweedii* | Less than 20 occurrences | | |
| *Idionycteris phyllotis* | INCLUDED | |  |
| *Irenomys tarsalis* | Not found north of equator | | |
| *Isolobodon montanus* | Less than 20 occurrences | | |
| *Isolobodon portoricensis* | Less than 20 occurrences | | |
| *Isothrix bistriata* | Less than 20 occurrences | | |
| *Isothrix negrensis* | Not found north of equator | | |
| *Isothrix pagurus* | Not found north of equator | | |
| *Isothrix sinnamariensis* | Less than 20 occurrences | | |
| *Isthmomys flavidus* | Less than 20 occurrences | | |
| *Isthmomys pirrensis* | Less than 20 occurrences | | |
| *Juliomys pictipes* | Not found north of equator | | |
| *Juliomys rimofrons* | Not found north of equator | | |
| *Juscelinomys guaporensis* | Not found north of equator | | |
| *Juscelinomys huanchacae* | Not found north of equator | | |
| *Kannabateomys amblyonyx* | Not found north of equator | | |
| *Kerodon acrobata* | Not found north of equator | | |
| *Kerodon rupestris* | Not found north of equator | | |
| *Kunsia fronto* | Not found north of equator | | |
| *Kunsia tomentosus* | Not found north of equator | | |
| *Lagidium peruanum* | Not found north of equator | | |
| *Lagidium viscacia* | Not found north of equator | | |
| *Lagidium wolffsohni* | Not found north of equator | | |
| *Lagothrix cana* | Not found north of equator | | |
| *Lagothrix lagothricha* | Less than 20 occurrences | | |
| *Lagothrix lugens* | INCLUDED | |  |
| *Lagostomus maximus* | Not found north of equator | | |
| *Lagothrix poeppigii* | Not found north of equator | | |
| *Lama lama* | Not found north of equator | | |
| *Lampronycteris brachyotis* | INCLUDED | |  |
| *Lasiurus blossevillii* | INCLUDED | |  |
| *Lasiurus borealis* | INCLUDED | |  |
| *Lasiurus castaneus* | INCLUDED | |  |
| *Lasiurus cinereus* | INCLUDED | |  |
| *Lasiurus degelidus* | Less than 20 occurrences | | |
| *Lasiurus ebenus* | Not found north of equator | | |
| *Lasiurus egregius* | INCLUDED | |  |
| *Lasiurus insularis* | Less than 20 occurrences | | |
| *Lasiurus intermedius* | INCLUDED | |  |
| *Lasiurus minor* | Less than 20 occurrences | | |
| *Lasionycteris noctivagans* | INCLUDED | |  |
| *Lasiurus pfeifferi* | Less than 20 occurrences | | |
| *Lasiurus seminolus* | INCLUDED | |  |
| *Lasiurus varius* | Not found north of equator | | |
| *Lasiurus xanthinus* | INCLUDED | |  |
| *Lemmiscus curtatus* | INCLUDED | |  |
| *Lemmus trimucronatus* | INCLUDED | |  |
| *Lenoxus apicalis* | Not found north of equator | | |
| *Leontopithecus caissara* | Not found north of equator | | |
| *Leontopithecus chrysomelas* | Not found north of equator | | |
| *Leontopithecus chrysopygus* | Not found north of equator | | |
| *Leontopithecus rosalia* | Not found north of equator | | |
| *Leopardus braccatus* | Not found north of equator | | |
| *Leopardus colocolo* | Not found north of equator | | |
| *Leopardus geoffroyi* | Not found north of equator | | |
| *Leopardus guigna* | Not found north of equator | | |
| *Leopardus jacobitus* | Not found north of equator | | |
| *Leopardus pajeros* | Not found north of equator | | |
| *Leopardus pardalis* | INCLUDED | |  |
| *Leopardus tigrinus* | INCLUDED | |  |
| *Leopardus wiedii* | INCLUDED | |  |
| *Leptonycteris curasoae* | INCLUDED | |  |
| *Leptonycteris nivalis* | INCLUDED | |  |
| *Leptonycteris yerbabuenae* | INCLUDED | |  |
| *Lepus alleni* | INCLUDED | |  |
| *Lepus americanus* | INCLUDED | |  |
| *Lepus arcticus* | INCLUDED | |  |
| *Lepus californicus* | INCLUDED | |  |
| *Lepus callotis* | INCLUDED | |  |
| *Lepus flavigularis* | Less than 20 occurrences | | |
| *Lepus insularis* | Less than 20 occurrences | | |
| *Lepus othus* | INCLUDED | |  |
| *Lepus townsendii* | INCLUDED | |  |
| *Lestodelphys halli* | Not found north of equator | | |
| *Lestoros inca* | Not found north of equator | | |
| *Lichonycteris obscura* | INCLUDED | |  |
| *Liomys adspersus* | Less than 20 occurrences | | |
| *Liomys irroratus* | INCLUDED | |  |
| *Liomys pictus* | INCLUDED | |  |
| *Liomys salvini* | INCLUDED | |  |
| *Liomys spectabilis* | Less than 20 occurrences | | |
| *Lionycteris spurrelli* | INCLUDED | |  |
| *Lonchorhina aurita* | INCLUDED | |  |
| *Lonchophylla bokermanni* | Not found north of equator | | |
| *Lonchophylla chocoana* | Not found north of equator | | |
| *Lonchophylla dekeyseri* | Not found north of equator | | |
| *Lonchothrix emiliae* | Not found north of equator | | |
| *Lonchorhina fernandezi* | Less than 20 occurrences | | |
| *Lonchophylla handleyi* | INCLUDED | |  |
| *Lonchophylla hesperia* | Not found north of equator | | |
| *Lonchorhina inusitata* | INCLUDED | |  |
| *Lonchorhina marinkellei* | Not found north of equator | | |
| *Lonchophylla mordax* | INCLUDED | |  |
| *Lonchorhina orinocensis* | INCLUDED | |  |
| *Lonchophylla robusta* | INCLUDED | |  |
| *Lonchophylla thomasi* | INCLUDED | |  |
| *Lontra canadensis* | INCLUDED | |  |
| *Lontra felina* | Not found north of equator | | |
| *Lontra longicaudis* | INCLUDED | |  |
| *Lontra provocax* | Not found north of equator | | |
| *Lophostoma brasiliense* | INCLUDED | |  |
| *Lophostoma carrikeri* | INCLUDED | |  |
| *Lophostoma evotis* | INCLUDED | |  |
| *Lophostoma schulzi* | Less than 20 occurrences | | |
| *Lophostoma silvicolum* | INCLUDED | |  |
| *Loxodontomys micropus* | Not found north of equator | | |
| *Loxodontomys pikumche* | Not found north of equator | | |
| *Lundomys molitor* | Not found north of equator | | |
| *Lutreolina crassicaudata* | INCLUDED | |  |
| *Lycalopex culpaeus* | Less than 20 occurrences | | |
| *Lycalopex fulvipes* | Not found north of equator | | |
| *Lycalopex griseus* | Not found north of equator | | |
| *Lycalopex gymnocercus* | Not found north of equator | | |
| *Lycalopex sechurae* | Not found north of equator | | |
| *Lycalopex vetulus* | Not found north of equator | | |
| *Lyncodon patagonicus* | Not found north of equator | | |
| *Lynx canadensis* | INCLUDED | |  |
| *Lynx rufus* | INCLUDED | |  |
| *Macrotus californicus* | INCLUDED | |  |
| *Macrophyllum macrophyllum* | INCLUDED | |  |
| *Macrotus waterhousii* | INCLUDED | |  |
| *Makalata didelphoides* | INCLUDED | |  |
| *Makalata grandis* | Not found north of equator | | |
| *Makalata macrura* | Not found north of equator | | |
| *Makalata occasius* | Not found north of equator | | |
| *Makalata rhipidura* | Not found north of equator | | |
| *Marmosa andersoni* | Not found north of equator | | |
| *Marmosops bishopi* | Not found north of equator | | |
| *Marmota broweri* | INCLUDED | |  |
| *Marmota caligata* | INCLUDED | |  |
| *Marmosops cracens* | Less than 20 occurrences | | |
| *Marmosops creightoni* | Not found north of equator | | |
| *Marmota flaviventris* | INCLUDED | |  |
| *Marmosops fuscatus* | INCLUDED | |  |
| *Marmosops impavidus* | Less than 20 occurrences | | |
| *Marmosops incanus* | Not found north of equator | | |
| *Marmosops invictus* | Less than 20 occurrences | | |
| *Marmosops juninensis* | Not found north of equator | | |
| *Marmosa lepida* | INCLUDED | |  |
| *Marmosa mexicana* | INCLUDED | |  |
| *Marmota monax* | INCLUDED | |  |
| *Marmosa murina* | INCLUDED | |  |
| *Marmosops neblina* | Not found north of equator | | |
| *Marmosops noctivagus* | Not found north of equator | | |
| *Marmosops ocellatus* | Not found north of equator | | |
| *Marmota olympus* | Less than 20 occurrences | | |
| *Marmosops parvidens* | INCLUDED | |  |
| *Marmosops paulensis* | Not found north of equator | | |
| *Marmosops pinheiroi* | INCLUDED | |  |
| *Marmosa robinsoni* | INCLUDED | |  |
| *Marmosa rubra* | Not found north of equator | | |
| *Marmosa tyleriana* | Less than 20 occurrences | | |
| *Marmota vancouverensis* | Less than 20 occurrences | | |
| *Marmosa xerophila* | Less than 20 occurrences | | |
| *Martes americana* | INCLUDED | |  |
| *Martes pennanti* | INCLUDED | |  |
| *Mazama americana* | INCLUDED | |  |
| *Mazama bricenii* | Less than 20 occurrences | | |
| *Mazama chunyi* | Not found north of equator | | |
| *Mazama gouazoubira* | INCLUDED | |  |
| *Mazama nana* | Not found north of equator | | |
| *Mazama pandora* | Less than 20 occurrences | | |
| *Mazama rufina* | Not found north of equator | | |
| *Megadontomys cryophilus* | Less than 20 occurrences | | |
| *Megasorex gigas* | INCLUDED | |  |
| *Megadontomys nelsoni* | Less than 20 occurrences | | |
| *Megadontomys thomasi* | Less than 20 occurrences | | |
| *Melanomys caliginosus* | INCLUDED | |  |
| *Melanomys robustulus* | Not found north of equator | | |
| *Melanomys zunigae* | Not found north of equator | | |
| *Mephitis macroura* | INCLUDED | |  |
| *Mephitis mephitis* | INCLUDED | |  |
| *Mesocapromys auritus* | Less than 20 occurrences | | |
| *Mesomys hispidus* | INCLUDED | |  |
| *Mesomys leniceps* | Not found north of equator | | |
| *Mesophylla macconnelli* | INCLUDED | |  |
| *Mesocapromys melanurus* | Less than 20 occurrences | | |
| *Mesocapromys nanus* | Less than 20 occurrences | | |
| *Mesomys stimulax* | Less than 20 occurrences | | |
| *Metachirus nudicaudatus* | INCLUDED | |  |
| *Micoureus alstoni* | INCLUDED | |  |
| *Micoureus constantiae* | Not found north of equator | | |
| *Micoureus demerarae* | INCLUDED | |  |
| *Micoureus paraguayanus* | Not found north of equator | | |
| *Micoureus regina* | Less than 20 occurrences | | |
| *Microsciurus alfari* | INCLUDED | |  |
| *Microryzomys altissimus* | Less than 20 occurrences | | |
| *Microcavia australis* | Not found north of equator | | |
| *Micronycteris brosseti* | Not found north of equator | | |
| *Microtus californicus* | INCLUDED | |  |
| *Microtus canicaudus* | Less than 20 occurrences | | |
| *Microtus chrotorrhinus* | INCLUDED | |  |
| *Microsciurus flaviventer* | INCLUDED | |  |
| *Microtus guatemalensis* | Less than 20 occurrences | | |
| *Micronycteris hirsuta* | INCLUDED | |  |
| *Micronycteris homezi* | Less than 20 occurrences | | |
| *Microtus longicaudus* | INCLUDED | |  |
| *Microdipodops megacephalus* | INCLUDED | |  |
| *Microtus mexicanus* | INCLUDED | |  |
| *Micronycteris microtis* | INCLUDED | |  |
| *Microsciurus mimulus* | INCLUDED | |  |
| *Microryzomys minutus* | INCLUDED | |  |
| *Microtus miurus* | INCLUDED | |  |
| *Microtus mogollonensis* | INCLUDED | |  |
| *Microtus montanus* | INCLUDED | |  |
| *Microcavia niata* | Not found north of equator | | |
| *Microtus oaxacensis* | Less than 20 occurrences | | |
| *Microtus ochrogaster* | INCLUDED | |  |
| *Microtus oeconomus* | INCLUDED | |  |
| *Microtus oregoni* | INCLUDED | |  |
| *Microdipodops pallidus* | Less than 20 occurrences | | |
| *Microtus pennsylvanicus* | INCLUDED | |  |
| *Microtus pinetorum* | INCLUDED | |  |
| *Microtus quasiater* | Less than 20 occurrences | | |
| *Microtus richardsoni* | INCLUDED | |  |
| *Micronycteris sanborni* | Not found north of equator | | |
| *Microsciurus santanderensis* | INCLUDED | |  |
| *Micronycteris schmidtorum* | INCLUDED | |  |
| *Microcavia shiptoni* | Not found north of equator | | |
| *Microtus townsendii* | INCLUDED | |  |
| *Microakodontomys transitorius* | Not found north of equator | | |
| *Microtus umbrosus* | Less than 20 occurrences | | |
| *Microtus xanthognathus* | INCLUDED | |  |
| *Mimon bennettii* | INCLUDED | |  |
| *Mimon cozumelae* | INCLUDED | |  |
| *Mimon crenulatum* | INCLUDED | |  |
| *Mimon koepckeae* | Not found north of equator | | |
| *Molossops aequatorianus* | Not found north of equator | | |
| *Molossus barnesi* | Less than 20 occurrences | | |
| *Molossus coibensis* | INCLUDED | |  |
| *Molossus currentium* | INCLUDED | |  |
| *Molossops mattogrossensis* | INCLUDED | |  |
| *Molossus molossus* | INCLUDED | |  |
| *Molossus pretiosus* | INCLUDED | |  |
| *Molossus rufus* | INCLUDED | |  |
| *Molossus sinaloae* | INCLUDED | |  |
| *Molossops temminckii* | INCLUDED | |  |
| *Monodelphis adusta* | Less than 20 occurrences | | |
| *Monodelphis americana* | Not found north of equator | | |
| *Monodelphis brevicaudata* | INCLUDED | |  |
| *Monodelphis dimidiata* | Not found north of equator | | |
| *Monodelphis domestica* | Not found north of equator | | |
| *Monodelphis emiliae* | Not found north of equator | | |
| *Monodelphis iheringi* | Not found north of equator | | |
| *Monodelphis kunsi* | Not found north of equator | | |
| *Monodelphis maraxina* | Not found north of equator | | |
| *Monodelphis osgoodi* | Not found north of equator | | |
| *Monophyllus plethodon* | Less than 20 occurrences | | |
| *Monophyllus redmani* | INCLUDED | |  |
| *Monodelphis rubida* | Not found north of equator | | |
| *Monodelphis scalops* | Not found north of equator | | |
| *Monodelphis sorex* | Not found north of equator | | |
| *Monodelphis theresa* | Not found north of equator | | |
| *Monodelphis unistriata* | Not found north of equator | | |
| *Mormoops blainvillii* | INCLUDED | |  |
| *Mormopterus kalinowskii* | Not found north of equator | | |
| *Mormoops megalophylla* | INCLUDED | |  |
| *Mormopterus minutus* | Less than 20 occurrences | | |
| *Mormopterus phrudus* | Not found north of equator | | |
| *Musonycteris harrisoni* | Less than 20 occurrences | | |
| *Mustela africana* | Less than 20 occurrences | | |
| *Mustela erminea* | INCLUDED | |  |
| *Mustela felipei* | Less than 20 occurrences | | |
| *Mustela frenata* | INCLUDED | |  |
| *Mustela nigripes* | INCLUDED | |  |
| *Mustela nivalis* | INCLUDED | |  |
| *Myocastor coypus* | INCLUDED | |  |
| *Myodes californicus* | INCLUDED | |  |
| *Myodes gapperi* | INCLUDED | |  |
| *Myodes rutilus* | INCLUDED | |  |
| *Myoprocta acouchy* | INCLUDED | |  |
| *Myoprocta pratti* | Less than 20 occurrences | | |
| *Myotis aelleni* | Not found north of equator | | |
| *Myotis albescens* | INCLUDED | |  |
| *Myotis atacamensis* | Not found north of equator | | |
| *Myotis auriculus* | INCLUDED | |  |
| *Myotis austroriparius* | INCLUDED | |  |
| *Myotis californicus* | INCLUDED | |  |
| *Myotis chiloensis* | Not found north of equator | | |
| *Myotis ciliolabrum* | INCLUDED | |  |
| *Myotis dominicensis* | Not found north of equator | | |
| *Myotis elegans* | INCLUDED | |  |
| *Myotis evotis* | INCLUDED | |  |
| *Myotis findleyi* | Not found north of equator | | |
| *Myotis fortidens* | INCLUDED | |  |
| *Myotis grisescens* | INCLUDED | |  |
| *Myotis keaysi* | INCLUDED | |  |
| *Myotis keenii* | INCLUDED | |  |
| *Myotis leibii* | INCLUDED | |  |
| *Myotis levis* | Not found north of equator | | |
| *Myotis lucifugus* | INCLUDED | |  |
| *Myotis martiniquensis* | Not found north of equator | | |
| *Myotis nesopolus* | Less than 20 occurrences | | |
| *Myotis nigricans* | INCLUDED | |  |
| *Myotis occultus* | INCLUDED | |  |
| *Myotis oxyotus* | INCLUDED | |  |
| *Myotis peninsularis* | Less than 20 occurrences | | |
| *Myotis planiceps* | Less than 20 occurrences | | |
| *Myotis riparius* | INCLUDED | |  |
| *Myotis ruber* | Not found north of equator | | |
| *Myotis septentrionalis* | INCLUDED | |  |
| *Myotis simus* | Less than 20 occurrences | | |
| *Myotis sodalis* | INCLUDED | |  |
| *Myotis thysanodes* | INCLUDED | |  |
| *Myotis velifer* | INCLUDED | |  |
| *Myotis vivesi* | INCLUDED | |  |
| *Myotis volans* | INCLUDED | |  |
| *Myotis yumanensis* | INCLUDED | |  |
| *Myrmecophaga tridactyla* | INCLUDED | |  |
| *Mysateles meridionalis* | Not found north of equator | | |
| *Mysateles prehensilis* | Less than 20 occurrences | | |
| *Napaeozapus insignis* | INCLUDED | |  |
| *Nasua narica* | INCLUDED | |  |
| *Nasua nasua* | INCLUDED | |  |
| *Nasuella olivacea* | INCLUDED | |  |
| *Natalus jamaicensis* | Less than 20 occurrences | | |
| *Natalus major* | Less than 20 occurrences | | |
| *Natalus primus* | Less than 20 occurrences | | |
| *Natalus stramineus* | INCLUDED | |  |
| *Neacomys dubosti* | Less than 20 occurrences | | |
| *Neacomys guianae* | INCLUDED | |  |
| *Neacomys minutus* | Not found north of equator | | |
| *Neacomys musseri* | Not found north of equator | | |
| *Neacomys paracou* | INCLUDED | |  |
| *Neacomys pictus* | Less than 20 occurrences | | |
| *Neacomys spinosus* | Less than 20 occurrences | | |
| *Neacomys tenuipes* | INCLUDED | |  |
| *Necromys amoenus* | Not found north of equator | | |
| *Necromys lactens* | Not found north of equator | | |
| *Necromys lasiurus* | Not found north of equator | | |
| *Necromys lenguarum* | Not found north of equator | | |
| *Necromys obscurus* | Not found north of equator | | |
| *Necromys punctulatus* | Not found north of equator | | |
| *Necromys temchuki* | Not found north of equator | | |
| *Necromys urichi* | INCLUDED | |  |
| *Nectomys apicalis* | Not found north of equator | | |
| *Nectomys magdalenae* | Less than 20 occurrences | | |
| *Nectomys palmipes* | INCLUDED | |  |
| *Nectomys rattus* | INCLUDED | |  |
| *Nectomys squamipes* | Not found north of equator | | |
| *Nelsonia goldmani* | Less than 20 occurrences | | |
| *Nelsonia neotomodon* | Less than 20 occurrences | | |
| *Neofiber alleni* | Less than 20 occurrences | | |
| *Neonycteris pusilla* | Not found north of equator | | |
| *Neotoma albigula* | INCLUDED | |  |
| *Neotamias alpinus* | Less than 20 occurrences | | |
| *Neotomodon alstoni* | Less than 20 occurrences | | |
| *Neotamias amoenus* | INCLUDED | |  |
| *Neotoma angustapalata* | Less than 20 occurrences | | |
| *Neotoma bryanti* | Not found north of equator | | |
| *Neotamias bulleri* | Less than 20 occurrences | | |
| *Neotamias canipes* | Less than 20 occurrences | | |
| *Neotoma chrysomelas* | Less than 20 occurrences | | |
| *Neotoma cinerea* | INCLUDED | |  |
| *Neotoma devia* | Less than 20 occurrences | | |
| *Neotamias dorsalis* | INCLUDED | |  |
| *Neotamias durangae* | Less than 20 occurrences | | |
| *Neotomys ebriosus* | Not found north of equator | | |
| *Neotoma floridana* | INCLUDED | |  |
| *Neotoma fuscipes* | INCLUDED | |  |
| *Neotoma goldmani* | INCLUDED | |  |
| *Neotoma lepida* | INCLUDED | |  |
| *Neotoma leucodon* | INCLUDED | |  |
| *Neotoma macrotis* | INCLUDED | |  |
| *Neotoma magister* | INCLUDED | |  |
| *Neotamias merriami* | INCLUDED | |  |
| *Neotoma mexicana* | INCLUDED | |  |
| *Neotoma micropus* | INCLUDED | |  |
| *Neotamias minimus* | INCLUDED | |  |
| *Neotoma nelsoni* | Less than 20 occurrences | | |
| *Neotamias obscurus* | Less than 20 occurrences | | |
| *Neotamias ochrogenys* | Less than 20 occurrences | | |
| *Neotoma palatina* | Less than 20 occurrences | | |
| *Neotamias palmeri* | Less than 20 occurrences | | |
| *Neotamias panamintinus* | Less than 20 occurrences | | |
| *Neotoma phenax* | Less than 20 occurrences | | |
| *Neotamias quadrimaculatus* | Less than 20 occurrences | | |
| *Neotamias quadrivittatus* | INCLUDED | |  |
| *Neotamias ruficaudus* | INCLUDED | |  |
| *Neotamias rufus* | INCLUDED | |  |
| *Neotamias senex* | INCLUDED | |  |
| *Neotamias siskiyou* | Less than 20 occurrences | | |
| *Neotamias sonomae* | Less than 20 occurrences | | |
| *Neotamias speciosus* | INCLUDED | |  |
| *Neotoma stephensi* | INCLUDED | |  |
| *Neotamias townsendii* | INCLUDED | |  |
| *Neotamias umbrinus* | INCLUDED | |  |
| *Neovison vison* | INCLUDED | |  |
| *Nesoryzomys darwini* | Not found north of equator | | |
| *Nesoryzomys fernandinae* | Not found north of equator | | |
| *Nesoryzomys indefessus* | Not found north of equator | | |
| *Nesoryzomys swarthi* | Not found north of equator | | |
| *Neurotrichus gibbsii* | INCLUDED | |  |
| *Neusticomys monticolus* | Less than 20 occurrences | | |
| *Neusticomys mussoi* | Less than 20 occurrences | | |
| *Neusticomys oyapocki* | Less than 20 occurrences | | |
| *Neusticomys peruviensis* | Not found north of equator | | |
| *Neusticomys venezuelae* | INCLUDED | |  |
| *Noctilio albiventris* | INCLUDED | |  |
| *Noctilio leporinus* | INCLUDED | |  |
| *Notiosorex crawfordi* | INCLUDED | |  |
| *Notiomys edwardsii* | Not found north of equator | | |
| *Notiosorex evotis* | INCLUDED | |  |
| *Notiosorex villai* | Less than 20 occurrences | | |
| *Nyctinomops aurispinosus* | INCLUDED | |  |
| *Nyctinomops femorosaccus* | INCLUDED | |  |
| *Nycticeius humeralis* | INCLUDED | |  |
| *Nyctinomops laticaudatus* | INCLUDED | |  |
| *Nyctiellus lepidus* | Less than 20 occurrences | | |
| *Nyctinomops macrotis* | INCLUDED | |  |
| *Nyctomys sumichrasti* | INCLUDED | |  |
| *Ochotona collaris* | INCLUDED | |  |
| *Ochotona princeps* | INCLUDED | |  |
| *Ochrotomys nuttalli* | INCLUDED | |  |
| *Octodon bridgesi* | Not found north of equator | | |
| *Octodon degus* | Not found north of equator | | |
| *Octodontomys gliroides* | Not found north of equator | | |
| *Octodon lunatus* | Not found north of equator | | |
| *Octomys mimax* | Not found north of equator | | |
| *Odocoileus hemionus* | INCLUDED | |  |
| *Odocoileus virginianus* | INCLUDED | |  |
| *Oecomys auyantepui* | INCLUDED | |  |
| *Oecomys bicolor* | INCLUDED | |  |
| *Oecomys cleberi* | Not found north of equator | | |
| *Oecomys concolor* | INCLUDED | |  |
| *Oecomys flavicans* | INCLUDED | |  |
| *Oecomys mamorae* | Not found north of equator | | |
| *Oecomys paricola* | Not found north of equator | | |
| *Oecomys phaeotis* | Not found north of equator | | |
| *Oecomys rex* | INCLUDED | |  |
| *Oecomys roberti* | INCLUDED | |  |
| *Oecomys rutilus* | INCLUDED | |  |
| *Oecomys speciosus* | INCLUDED | |  |
| *Oecomys superans* | Less than 20 occurrences | | |
| *Oecomys trinitatis* | INCLUDED | |  |
| *Olallamys albicauda* | Less than 20 occurrences | | |
| *Olallamys edax* | Less than 20 occurrences | | |
| *Oligoryzomys andinus* | Not found north of equator | | |
| *Oligoryzomys arenalis* | Not found north of equator | | |
| *Oligoryzomys chacoensis* | Not found north of equator | | |
| *Oligoryzomys delticola* | Not found north of equator | | |
| *Oligoryzomys destructor* | Less than 20 occurrences | | |
| *Oligoryzomys eliurus* | Not found north of equator | | |
| *Oligoryzomys flavescens* | Not found north of equator | | |
| *Oligoryzomys fulvescens* | INCLUDED | |  |
| *Oligoryzomys griseolus* | Less than 20 occurrences | | |
| *Oligoryzomys longicaudatus* | Not found north of equator | | |
| *Oligoryzomys magellanicus* | Not found north of equator | | |
| *Oligoryzomys microtis* | Not found north of equator | | |
| *Oligoryzomys nigripes* | Not found north of equator | | |
| *Oligoryzomys stramineus* | Not found north of equator | | |
| *Oligoryzomys vegetus* | Less than 20 occurrences | | |
| *Oligoryzomys victus* | Not found north of equator | | |
| *Ondatra zibethicus* | INCLUDED | |  |
| *Onychomys arenicola* | INCLUDED | |  |
| *Onychomys leucogaster* | INCLUDED | |  |
| *Onychomys torridus* | INCLUDED | |  |
| *Oreamnos americanus* | INCLUDED | |  |
| *Oreonax flavicauda* | Not found north of equator | | |
| *Orthogeomys cavator* | Less than 20 occurrences | | |
| *Orthogeomys cherriei* | Less than 20 occurrences | | |
| *Orthogeomys cuniculus* | Less than 20 occurrences | | |
| *Orthogeomys dariensis* | Less than 20 occurrences | | |
| *Orthogeomys grandis* | INCLUDED | |  |
| *Orthogeomys heterodus* | Less than 20 occurrences | | |
| *Orthogeomys hispidus* | INCLUDED | |  |
| *Orthogeomys lanius* | Less than 20 occurrences | | |
| *Orthogeomys matagalpae* | Less than 20 occurrences | | |
| *Orthogeomys thaeleri* | Less than 20 occurrences | | |
| *Orthogeomys underwoodi* | Less than 20 occurrences | | |
| *Oryzomys albigularis* | INCLUDED | |  |
| *Oryzomys alfaroi* | INCLUDED | |  |
| *Oryzomys angouya* | Not found north of equator | | |
| *Oryzomys auriventer* | Not found north of equator | | |
| *Oryzomys balneator* | Not found north of equator | | |
| *Oryzomys bolivaris* | INCLUDED | |  |
| *Oryzomys chapmani* | INCLUDED | |  |
| *Oryzomys couesi* | INCLUDED | |  |
| *Oryzomys devius* | Less than 20 occurrences | | |
| *Oryzomys dimidiatus* | Less than 20 occurrences | | |
| *Oryzomys emmonsae* | Not found north of equator | | |
| *Oryzomys galapagoensis* | Not found north of equator | | |
| *Oryzomys gorgasi* | Less than 20 occurrences | | |
| *Oryzomys hammondi* | Not found north of equator | | |
| *Oryzomys keaysi* | Not found north of equator | | |
| *Oryzomys laticeps* | Not found north of equator | | |
| *Oryzomys levipes* | Not found north of equator | | |
| *Oryzomys macconnelli* | INCLUDED | |  |
| *Oryzomys megacephalus* | Less than 20 occurrences | | |
| *Oryzomys melanotis* | INCLUDED | |  |
| *Oryzomys nelsoni* | Not found north of equator | | |
| *Oryzomys nitidus* | Not found north of equator | | |
| *Oryzomys palustris* | INCLUDED | |  |
| *Oryzomys perenensis* | Not found north of equator | | |
| *Oryzomys polius* | Not found north of equator | | |
| *Oryzomys rhabdops* | Less than 20 occurrences | | |
| *Oryzomys rostratus* | INCLUDED | |  |
| *Oryzomys russatus* | Not found north of equator | | |
| *Oryzomys saturatior* | INCLUDED | |  |
| *Oryzomys seuanezi* | Not found north of equator | | |
| *Oryzomys subflavus* | Not found north of equator | | |
| *Oryzomys talamancae* | INCLUDED | |  |
| *Oryzomys tatei* | Not found north of equator | | |
| *Oryzomys xanthaeolus* | Not found north of equator | | |
| *Oryzomys yunganus* | INCLUDED | |  |
| *Osgoodomys banderanus* | INCLUDED | |  |
| *Otonyctomys hatti* | Less than 20 occurrences | | |
| *Ototylomys phyllotis* | INCLUDED | |  |
| *Ovibos moschatus* | INCLUDED | |  |
| *Ovis canadensis* | INCLUDED | |  |
| *Ovis dalli* | INCLUDED | |  |
| *Oxymycterus akodontius* | Not found north of equator | | |
| *Oxymycterus amazonicus* | Not found north of equator | | |
| *Oxymycterus angularis* | Not found north of equator | | |
| *Oxymycterus caparaoe* | Not found north of equator | | |
| *Oxymycterus hiska* | Not found north of equator | | |
| *Oxymycterus hispidus* | Not found north of equator | | |
| *Oxymycterus hucucha* | Not found north of equator | | |
| *Oxymycterus inca* | Not found north of equator | | |
| *Oxymycterus josei* | Not found north of equator | | |
| *Oxymycterus nasutus* | Not found north of equator | | |
| *Oxymycterus paramensis* | Not found north of equator | | |
| *Oxymycterus roberti* | Not found north of equator | | |
| *Oxymycterus rufus* | Not found north of equator | | |
| *Ozotoceros bezoarticus* | Not found north of equator | | |
| *Panthera onca* | INCLUDED | |  |
| *Pappogeomys alcorni* | Less than 20 occurrences | | |
| *Pappogeomys bulleri* | Less than 20 occurrences | | |
| *Parascalops breweri* | INCLUDED | |  |
| *Paralomys gerbillus* | Not found north of equator | | |
| *Pearsonomys annectens* | Not found north of equator | | |
| *Pecari tajacu* | INCLUDED | |  |
| *Perimyotis subflavus* | INCLUDED | |  |
| *Perognathus alticolus* | Less than 20 occurrences | | |
| *Perognathus amplus* | INCLUDED | |  |
| *Peromyscus attwateri* | INCLUDED | |  |
| *Peromyscus aztecus* | Less than 20 occurrences | | |
| *Peromyscus beatae* | INCLUDED | |  |
| *Peromyscus boylii* | INCLUDED | |  |
| *Peromyscus bullatus* | Less than 20 occurrences | | |
| *Peromyscus californicus* | INCLUDED | |  |
| *Peromyscus crinitus* | INCLUDED | |  |
| *Peromyscus difficilis* | INCLUDED | |  |
| *Peromyscus eremicus* | INCLUDED | |  |
| *Peromyscus eva* | Less than 20 occurrences | | |
| *Perognathus fasciatus* | INCLUDED | |  |
| *Perognathus flavescens* | INCLUDED | |  |
| *Perognathus flavus* | INCLUDED | |  |
| *Peromyscus fraterculus* | INCLUDED | |  |
| *Peromyscus furvus* | Less than 20 occurrences | | |
| *Peromyscus gossypinus* | INCLUDED | |  |
| *Peromyscus grandis* | Less than 20 occurrences | | |
| *Peromyscus gratus* | INCLUDED | |  |
| *Peromyscus guardia* | Less than 20 occurrences | | |
| *Peromyscus guatemalensis* | Less than 20 occurrences | | |
| *Peromyscus gymnotis* | Less than 20 occurrences | | |
| *Peromyscus hooperi* | Less than 20 occurrences | | |
| *Perognathus inornatus* | Less than 20 occurrences | | |
| *Peropteryx kappleri* | INCLUDED | |  |
| *Peromyscus keeni* | INCLUDED | |  |
| *Peromyscus leucopus* | INCLUDED | |  |
| *Peromyscus levipes* | INCLUDED | |  |
| *Perognathus longimembris* | INCLUDED | |  |
| *Peropteryx macrotis* | INCLUDED | |  |
| *Peromyscus madrensis* | Not found north of equator | | |
| *Peromyscus maniculatus* | INCLUDED | |  |
| *Peromyscus mayensis* | Less than 20 occurrences | | |
| *Peromyscus megalops* | Less than 20 occurrences | | |
| *Peromyscus mekisturus* | Less than 20 occurrences | | |
| *Peromyscus melanocarpus* | Less than 20 occurrences | | |
| *Peromyscus melanophrys* | INCLUDED | |  |
| *Peromyscus melanotis* | INCLUDED | |  |
| *Peromyscus melanurus* | Less than 20 occurrences | | |
| *Peromyscus merriami* | INCLUDED | |  |
| *Peromyscus mexicanus* | INCLUDED | |  |
| *Peromyscus nasutus* | INCLUDED | |  |
| *Peromyscus ochraventer* | Less than 20 occurrences | | |
| *Perognathus parvus* | INCLUDED | |  |
| *Peromyscus pectoralis* | INCLUDED | |  |
| *Peromyscus perfulvus* | Less than 20 occurrences | | |
| *Peromyscus polionotus* | INCLUDED | |  |
| *Peromyscus polius* | Less than 20 occurrences | | |
| *Peromyscus sagax* | Less than 20 occurrences | | |
| *Peromyscus schmidlyi* | Less than 20 occurrences | | |
| *Peromyscus simulus* | Less than 20 occurrences | | |
| *Peromyscus spicilegus* | INCLUDED | |  |
| *Peromyscus stirtoni* | Less than 20 occurrences | | |
| *Peromyscus truei* | INCLUDED | |  |
| *Peromyscus winkelmanni* | Less than 20 occurrences | | |
| *Peromyscus yucatanicus* | Less than 20 occurrences | | |
| *Peromyscus zarhynchus* | Less than 20 occurrences | | |
| *Phaenomys ferrugineus* | Not found north of equator | | |
| *Phenacomys intermedius* | INCLUDED | |  |
| *Phenacomys ungava* | INCLUDED | |  |
| *Philander andersoni* | Less than 20 occurrences | | |
| *Philander frenatus* | Not found north of equator | | |
| *Philander mcilhennyi* | Not found north of equator | | |
| *Philander opossum* | INCLUDED | |  |
| *Phyllotis amicus* | Not found north of equator | | |
| *Phyllotis andium* | Not found north of equator | | |
| *Phyllonycteris aphylla* | Less than 20 occurrences | | |
| *Phyllomys blainvilii* | Not found north of equator | | |
| *Phyllotis bonariensis* | Not found north of equator | | |
| *Phyllomys brasiliensis* | Not found north of equator | | |
| *Phyllotis caprinus* | Not found north of equator | | |
| *Phyllotis darwini* | Not found north of equator | | |
| *Phyllomys dasythrix* | Not found north of equator | | |
| *Phyllotis definitus* | Not found north of equator | | |
| *Phyllostomus discolor* | INCLUDED | |  |
| *Phyllostomus elongatus* | INCLUDED | |  |
| *Phyllops falcatus* | INCLUDED | |  |
| *Phyllotis haggardi* | Not found north of equator | | |
| *Phyllostomus hastatus* | INCLUDED | |  |
| *Phyllomys kerri* | Not found north of equator | | |
| *Phyllomys lamarum* | Not found north of equator | | |
| *Phyllostomus latifolius* | INCLUDED | |  |
| *Phyllotis limatus* | Not found north of equator | | |
| *Phyllomys lundi* | Not found north of equator | | |
| *Phyllotis magister* | Not found north of equator | | |
| *Phyllomys medius* | Not found north of equator | | |
| *Phyllomys nigrispinus* | Not found north of equator | | |
| *Phyllotis osgoodi* | Not found north of equator | | |
| *Phyllotis osilae* | Not found north of equator | | |
| *Phyllomys pattoni* | Not found north of equator | | |
| *Phyllonycteris poeyi* | INCLUDED | |  |
| *Phylloderma stenops* | INCLUDED | |  |
| *Phyllomys thomasi* | Not found north of equator | | |
| *Phyllotis wolffsohni* | Not found north of equator | | |
| *Phyllotis xanthopygus* | Not found north of equator | | |
| *Pipistrellus hesperus* | INCLUDED | |  |
| *Pithecia aequatorialis* | Not found north of equator | | |
| *Pithecia albicans* | Not found north of equator | | |
| *Pithecia irrorata* | Not found north of equator | | |
| *Pithecia monachus* | Less than 20 occurrences | | |
| *Pithecia pithecia* | INCLUDED | |  |
| *Plagiodontia aedium* | Less than 20 occurrences | | |
| *Plagiodontia araeum* | Less than 20 occurrences | | |
| *Plagiodontia ipnaeum* | Less than 20 occurrences | | |
| *Platyrrhinus aurarius* | INCLUDED | |  |
| *Platyrrhinus brachycephalus* | INCLUDED | |  |
| *Platyrrhinus chocoensis* | Less than 20 occurrences | | |
| *Platyrrhinus dorsalis* | INCLUDED | |  |
| *Platalina genovensium* | Not found north of equator | | |
| *Platyrrhinus helleri* | INCLUDED | |  |
| *Platyrrhinus infuscus* | INCLUDED | |  |
| *Platyrrhinus lineatus* | INCLUDED | |  |
| *Platyrrhinus recifinus* | INCLUDED | |  |
| *Platyrrhinus umbratus* | Less than 20 occurrences | | |
| *Platyrrhinus vittatus* | INCLUDED | |  |
| *Podomys floridanus* | Less than 20 occurrences | | |
| *Podoxymys roraimae* | Less than 20 occurrences | | |
| *Potos flavus* | INCLUDED | |  |
| *Priodontes maximus* | INCLUDED | |  |
| *Procyon cancrivorus* | INCLUDED | |  |
| *Procyon lotor* | INCLUDED | |  |
| *Procyon pygmaeus* | Less than 20 occurrences | | |
| *Proechimys brevicauda* | Less than 20 occurrences | | |
| *Proechimys canicollis* | Less than 20 occurrences | | |
| *Proechimys chrysaeolus* | Less than 20 occurrences | | |
| *Proechimys cuvieri* | INCLUDED | |  |
| *Proechimys decumanus* | Not found north of equator | | |
| *Proechimys echinothrix* | Not found north of equator | | |
| *Proechimys gardneri* | Not found north of equator | | |
| *Proechimys goeldii* | Not found north of equator | | |
| *Proechimys guairae* | INCLUDED | |  |
| *Proechimys guyannensis* | INCLUDED | |  |
| *Proechimys hoplomyoides* | INCLUDED | |  |
| *Proechimys kulinae* | Not found north of equator | | |
| *Proechimys longicaudatus* | Not found north of equator | | |
| *Proechimys magdalenae* | INCLUDED | |  |
| *Proechimys mincae* | Less than 20 occurrences | | |
| *Proechimys oconnelli* | Less than 20 occurrences | | |
| *Proechimys pattoni* | Not found north of equator | | |
| *Proechimys poliopus* | Less than 20 occurrences | | |
| *Proechimys quadruplicatus* | Less than 20 occurrences | | |
| *Proechimys roberti* | Not found north of equator | | |
| *Proechimys semispinosus* | Less than 20 occurrences | | |
| *Proechimys simonsi* | Less than 20 occurrences | | |
| *Proechimys steerei* | Not found north of equator | | |
| *Proechimys trinitatis* | Less than 20 occurrences | | |
| *Proechimys urichi* | Less than 20 occurrences | | |
| *Promops centralis* | INCLUDED | |  |
| *Promops nasutus* | INCLUDED | |  |
| *Pseudoryzomys simplex* | Not found north of equator | | |
| *Pteronura brasiliensis* | INCLUDED | |  |
| *Pteronotus davyi* | INCLUDED | |  |
| *Pteronotus gymnonotus* | INCLUDED | |  |
| *Pteronotus macleayii* | Less than 20 occurrences | | |
| *Pteronotus parnellii* | INCLUDED | |  |
| *Pteronotus personatus* | INCLUDED | |  |
| *Pteronotus quadridens* | INCLUDED | |  |
| *Pudu mephistophiles* | Less than 20 occurrences | | |
| *Pudu puda* | Not found north of equator | | |
| *Puma concolor* | INCLUDED | |  |
| *Puma yagouaroundi* | INCLUDED | |  |
| *Punomys kofordi* | Not found north of equator | | |
| *Punomys lemminus* | Not found north of equator | | |
| *Pygoderma bilabiatum* | Less than 20 occurrences | | |
| *Rangifer tarandus* | INCLUDED | |  |
| *Reithrodon auritus* | Not found north of equator | | |
| *Reithrodontomys bakeri* | Less than 20 occurrences | | |
| *Reithrodontomys brevirostris* | Less than 20 occurrences | | |
| *Reithrodontomys burti* | Less than 20 occurrences | | |
| *Reithrodontomys chrysopsis* | Less than 20 occurrences | | |
| *Reithrodontomys creper* | Less than 20 occurrences | | |
| *Reithrodontomys darienensis* | Less than 20 occurrences | | |
| *Reithrodontomys fulvescens* | INCLUDED | |  |
| *Reithrodontomys gracilis* | INCLUDED | |  |
| *Reithrodontomys hirsutus* | Less than 20 occurrences | | |
| *Reithrodontomys humulis* | INCLUDED | |  |
| *Reithrodontomys megalotis* | INCLUDED | |  |
| *Reithrodontomys mexicanus* | INCLUDED | |  |
| *Reithrodontomys microdon* | INCLUDED | |  |
| *Reithrodontomys montanus* | INCLUDED | |  |
| *Reithrodontomys paradoxus* | Less than 20 occurrences | | |
| *Reithrodontomys raviventris* | Less than 20 occurrences | | |
| *Reithrodontomys rodriguezi* | Less than 20 occurrences | | |
| *Reithrodontomys spectabilis* | Less than 20 occurrences | | |
| *Reithrodontomys sumichrasti* | INCLUDED | |  |
| *Reithrodontomys tenuirostris* | Less than 20 occurrences | | |
| *Reithrodontomys zacatecae* | INCLUDED | |  |
| *Rhagomys longilingua* | Not found north of equator | | |
| *Rheomys mexicanus* | Less than 20 occurrences | | |
| *Rheomys raptor* | Less than 20 occurrences | | |
| *Rheomys thomasi* | Less than 20 occurrences | | |
| *Rheomys underwoodi* | Less than 20 occurrences | | |
| *Rhinophylla alethina* | Less than 20 occurrences | | |
| *Rhinophylla fischerae* | Less than 20 occurrences | | |
| *Rhinophylla pumilio* | INCLUDED | |  |
| *Rhipidomys austrinus* | Not found north of equator | | |
| *Rhipidomys caucensis* | Less than 20 occurrences | | |
| *Rhipidomys couesi* | INCLUDED | |  |
| *Rhipidomys fulviventer* | Less than 20 occurrences | | |
| *Rhipidomys gardneri* | Not found north of equator | | |
| *Rhipidomys latimanus* | INCLUDED | |  |
| *Rhipidomys leucodactylus* | INCLUDED | |  |
| *Rhipidomys macconnelli* | INCLUDED | |  |
| *Rhipidomys mastacalis* | Not found north of equator | | |
| *Rhipidomys nitela* | INCLUDED | |  |
| *Rhipidomys ochrogaster* | Not found north of equator | | |
| *Rhipidomys venezuelae* | INCLUDED | |  |
| *Rhipidomys venustus* | INCLUDED | |  |
| *Rhipidomys wetzeli* | INCLUDED | |  |
| *Rhogeessa aeneus* | Less than 20 occurrences | | |
| *Rhogeessa alleni* | INCLUDED | |  |
| *Rhogeessa genowaysi* | Less than 20 occurrences | | |
| *Rhogeessa gracilis* | INCLUDED | |  |
| *Rhogeessa minutilla* | INCLUDED | |  |
| *Rhogeessa mira* | Less than 20 occurrences | | |
| *Rhogeessa parvula* | INCLUDED | |  |
| *Rhogeessa tumida* | INCLUDED | |  |
| *Rhynchonycteris naso* | INCLUDED | |  |
| *Rhyncholestes raphanurus* | Not found north of equator | | |
| *Romerolagus diazi* | Less than 20 occurrences | | |
| *Saccopteryx bilineata* | INCLUDED | |  |
| *Saccopteryx canescens* | INCLUDED | |  |
| *Saccopteryx gymnura* | Less than 20 occurrences | | |
| *Saccopteryx leptura* | INCLUDED | |  |
| *Saguinus bicolor* | Not found north of equator | | |
| *Saguinus fuscicollis* | Not found north of equator | | |
| *Saguinus geoffroyi* | Less than 20 occurrences | | |
| *Saguinus graellsi* | Not found north of equator | | |
| *Saguinus imperator* | Not found north of equator | | |
| *Saguinus inustus* | Less than 20 occurrences | | |
| *Saguinus labiatus* | Not found north of equator | | |
| *Saguinus leucopus* | Less than 20 occurrences | | |
| *Saguinus martinsi* | Not found north of equator | | |
| *Saguinus midas* | INCLUDED | |  |
| *Saguinus mystax* | Not found north of equator | | |
| *Saguinus niger* | Not found north of equator | | |
| *Saguinus nigricollis* | Less than 20 occurrences | | |
| *Saguinus oedipus* | Less than 20 occurrences | | |
| *Saguinus tripartitus* | Not found north of equator | | |
| *Saimiri boliviensis* | Not found north of equator | | |
| *Saimiri oerstedii* | Less than 20 occurrences | | |
| *Saimiri sciureus* | INCLUDED | |  |
| *Saimiri ustus* | Not found north of equator | | |
| *Saimiri vanzolinii* | Not found north of equator | | |
| *Salinomys delicatus* | Not found north of equator | | |
| *Salinoctomys loschalchalerosorum* | Not found north of equator | | |
| *Scalopus aquaticus* | INCLUDED | |  |
| *Scapteromys aquaticus* | Not found north of equator | | |
| *Scapanus latimanus* | INCLUDED | |  |
| *Scapanus orarius* | INCLUDED | |  |
| *Scapanus townsendii* | INCLUDED | |  |
| *Scapteromys tumidus* | Not found north of equator | | |
| *Sciurus aberti* | INCLUDED | |  |
| *Sciurus aestuans* | INCLUDED | |  |
| *Sciurus alleni* | Less than 20 occurrences | | |
| *Sciurus arizonensis* | Less than 20 occurrences | | |
| *Sciurus aureogaster* | INCLUDED | |  |
| *Sciurus carolinensis* | INCLUDED | |  |
| *Sciurus colliaei* | INCLUDED | |  |
| *Sciurus deppei* | INCLUDED | |  |
| *Sciurus flammifer* | INCLUDED | |  |
| *Sciurus gilvigularis* | Less than 20 occurrences | | |
| *Sciurus granatensis* | INCLUDED | |  |
| *Sciurus griseus* | INCLUDED | |  |
| *Sciurus ignitus* | Not found north of equator | | |
| *Sciurus igniventris* | INCLUDED | |  |
| *Sciurus nayaritensis* | INCLUDED | |  |
| *Sciurus niger* | INCLUDED | |  |
| *Sciurus oculatus* | INCLUDED | |  |
| *Sciurus pucheranii* | INCLUDED | |  |
| *Sciurillus pusillus* | INCLUDED | |  |
| *Sciurus pyrrhinus* | Not found north of equator | | |
| *Sciurus richmondi* | Less than 20 occurrences | | |
| *Sciurus sanborni* | Not found north of equator | | |
| *Sciurus spadiceus* | Less than 20 occurrences | | |
| *Sciurus stramineus* | Not found north of equator | | |
| *Sciurus variegatoides* | INCLUDED | |  |
| *Sciurus yucatanensis* | INCLUDED | |  |
| *Scleronycteris ega* | Less than 20 occurrences | | |
| *Scolomys melanops* | Not found north of equator | | |
| *Scolomys ucayalensis* | Not found north of equator | | |
| *Scotinomys teguina* | INCLUDED | |  |
| *Scotinomys xerampelinus* | Less than 20 occurrences | | |
| *Sigmodontomys alfari* | INCLUDED | |  |
| *Sigmodon alleni* | INCLUDED | |  |
| *Sigmodon alstoni* | INCLUDED | |  |
| *Sigmodontomys aphrastus* | Less than 20 occurrences | | |
| *Sigmodon arizonae* | INCLUDED | |  |
| *Sigmodon fulviventer* | INCLUDED | |  |
| *Sigmodon hispidus* | INCLUDED | |  |
| *Sigmodon inopinatus* | Not found north of equator | | |
| *Sigmodon leucotis* | INCLUDED | |  |
| *Sigmodon mascotensis* | INCLUDED | |  |
| *Sigmodon ochrognathus* | INCLUDED | |  |
| *Sigmodon peruanus* | Not found north of equator | | |
| *Solenodon cubanus* | Less than 20 occurrences | | |
| *Solenodon paradoxus* | Less than 20 occurrences | | |
| *Sorex alaskanus* | Less than 20 occurrences | | |
| *Sorex arcticus* | INCLUDED | |  |
| *Sorex arizonae* | Less than 20 occurrences | | |
| *Sorex bairdi* | Less than 20 occurrences | | |
| *Sorex bendirii* | INCLUDED | |  |
| *Sorex cinereus* | INCLUDED | |  |
| *Sorex dispar* | INCLUDED | |  |
| *Sorex emarginatus* | Less than 20 occurrences | | |
| *Sorex fontinalis* | Less than 20 occurrences | | |
| *Sorex fumeus* | INCLUDED | |  |
| *Sorex gaspensis* | Less than 20 occurrences | | |
| *Sorex haydeni* | INCLUDED | |  |
| *Sorex hoyi* | INCLUDED | |  |
| *Sorex longirostris* | INCLUDED | |  |
| *Sorex lyelli* | Less than 20 occurrences | | |
| *Sorex macrodon* | Less than 20 occurrences | | |
| *Sorex merriami* | INCLUDED | |  |
| *Sorex milleri* | Less than 20 occurrences | | |
| *Sorex monticolus* | INCLUDED | |  |
| *Sorex nanus* | INCLUDED | |  |
| *Sorex neomexicanus* | Less than 20 occurrences | | |
| *Sorex oreopolus* | Less than 20 occurrences | | |
| *Sorex ornatus* | INCLUDED | |  |
| *Sorex pacificus* | INCLUDED | |  |
| *Sorex palustris* | INCLUDED | |  |
| *Sorex preblei* | INCLUDED | |  |
| *Sorex rohweri* | Less than 20 occurrences | | |
| *Sorex saussurei* | INCLUDED | |  |
| *Sorex sclateri* | Less than 20 occurrences | | |
| *Sorex sonomae* | INCLUDED | |  |
| *Sorex stizodon* | Less than 20 occurrences | | |
| *Sorex tenellus* | Less than 20 occurrences | | |
| *Sorex trowbridgii* | INCLUDED | |  |
| *Sorex tundrensis* | INCLUDED | |  |
| *Sorex ugyunak* | INCLUDED | |  |
| *Sorex vagrans* | INCLUDED | |  |
| *Sorex ventralis* | INCLUDED | |  |
| *Sorex veraepacis* | Less than 20 occurrences | | |
| *Spalacopus cyanus* | Not found north of equator | | |
| *Speothos venaticus* | INCLUDED | |  |
| *Spermophilus adocetus* | Less than 20 occurrences | | |
| *Spermophilus annulatus* | Less than 20 occurrences | | |
| *Spermophilus armatus* | INCLUDED | |  |
| *Spermophilus atricapillus* | Less than 20 occurrences | | |
| *Spermophilus beecheyi* | INCLUDED | |  |
| *Spermophilus beldingi* | INCLUDED | |  |
| *Spermophilus brunneus* | Less than 20 occurrences | | |
| *Spermophilus canus* | INCLUDED | |  |
| *Spermophilus columbianus* | INCLUDED | |  |
| *Spermophilus elegans* | INCLUDED | |  |
| *Spermophilus franklinii* | INCLUDED | |  |
| *Spermophilus lateralis* | INCLUDED | |  |
| *Spermophilus madrensis* | Less than 20 occurrences | | |
| *Spermophilus mexicanus* | INCLUDED | |  |
| *Spermophilus mohavensis* | Less than 20 occurrences | | |
| *Spermophilus mollis* | INCLUDED | |  |
| *Spermophilus parryii* | INCLUDED | |  |
| *Spermophilus perotensis* | Less than 20 occurrences | | |
| *Spermophilus richardsonii* | INCLUDED | |  |
| *Spermophilus saturatus* | INCLUDED | |  |
| *Spermophilus spilosoma* | INCLUDED | |  |
| *Spermophilus tereticaudus* | INCLUDED | |  |
| *Spermophilus townsendii* | Less than 20 occurrences | | |
| *Spermophilus tridecemlineatus* | INCLUDED | |  |
| *Spermophilus variegatus* | INCLUDED | |  |
| *Spermophilus washingtoni* | Less than 20 occurrences | | |
| *Sphaeronycteris toxophyllum* | INCLUDED | |  |
| *Sphiggurus ichillus* | Not found north of equator | | |
| *Sphiggurus insidiosus* | Not found north of equator | | |
| *Sphiggurus melanurus* | INCLUDED | |  |
| *Sphiggurus mexicanus* | INCLUDED | |  |
| *Sphiggurus pruinosus* | INCLUDED | |  |
| *Sphiggurus roosmalenorum* | Not found north of equator | | |
| *Sphiggurus spinosus* | Not found north of equator | | |
| *Sphiggurus vestitus* | Less than 20 occurrences | | |
| *Sphiggurus villosus* | Not found north of equator | | |
| *Spilogale gracilis* | INCLUDED | |  |
| *Spilogale putorius* | INCLUDED | |  |
| *Spilogale pygmaea* | INCLUDED | |  |
| *Stenoderma rufum* | Less than 20 occurrences | | |
| *Sturnira aratathomasi* | Less than 20 occurrences | | |
| *Sturnira bidens* | INCLUDED | |  |
| *Sturnira bogotensis* | INCLUDED | |  |
| *Sturnira erythromos* | INCLUDED | |  |
| *Sturnira koopmanhilli* | Less than 20 occurrences | | |
| *Sturnira lilium* | INCLUDED | |  |
| *Sturnira ludovici* | INCLUDED | |  |
| *Sturnira luisi* | INCLUDED | |  |
| *Sturnira magna* | Less than 20 occurrences | | |
| *Sturnira mordax* | Less than 20 occurrences | | |
| *Sturnira nana* | Not found north of equator | | |
| *Sturnira oporaphilum* | Not found north of equator | | |
| *Sturnira thomasi* | Less than 20 occurrences | | |
| *Sturnira tildae* | INCLUDED | |  |
| *Sylvilagus aquaticus* | INCLUDED | |  |
| *Sylvilagus audubonii* | INCLUDED | |  |
| *Sylvilagus bachmani* | INCLUDED | |  |
| *Sylvilagus brasiliensis* | INCLUDED | |  |
| *Sylvilagus cognatus* | Less than 20 occurrences | | |
| *Sylvilagus cunicularius* | INCLUDED | |  |
| *Sylvilagus dicei* | Less than 20 occurrences | | |
| *Sylvilagus floridanus* | INCLUDED | |  |
| *Sylvilagus graysoni* | Not found north of equator | | |
| *Sylvilagus insonus* | Less than 20 occurrences | | |
| *Sylvilagus mansuetus* | Less than 20 occurrences | | |
| *Sylvilagus nuttallii* | INCLUDED | |  |
| *Sylvilagus obscurus* | INCLUDED | |  |
| *Sylvilagus palustris* | INCLUDED | |  |
| *Sylvilagus robustus* | Less than 20 occurrences | | |
| *Sylvilagus transitionalis* | INCLUDED | |  |
| *Synaptomys borealis* | INCLUDED | |  |
| *Synaptomys cooperi* | INCLUDED | |  |
| *Syntheosciurus brochus* | Less than 20 occurrences | | |
| *Tadarida brasiliensis* | INCLUDED | |  |
| *Tamandua mexicana* | INCLUDED | |  |
| *Tamandua tetradactyla* | INCLUDED | |  |
| *Tamiasciurus douglasii* | INCLUDED | |  |
| *Tamiasciurus hudsonicus* | INCLUDED | |  |
| *Tamiasciurus mearnsi* | Less than 20 occurrences | | |
| *Tamias striatus* | INCLUDED | |  |
| *Tapecomys primus* | Not found north of equator | | |
| *Tapirus bairdii* | INCLUDED | |  |
| *Tapirus pinchaque* | INCLUDED | |  |
| *Tapirus terrestris* | INCLUDED | |  |
| *Taxidea taxus* | INCLUDED | |  |
| *Tayassu pecari* | INCLUDED | |  |
| *Thalpomys cerradensis* | Not found north of equator | | |
| *Thalpomys lasiotis* | Not found north of equator | | |
| *Thaptomys nigrita* | Not found north of equator | | |
| *Thomasomys apeco* | Not found north of equator | | |
| *Thomasomys aureus* | INCLUDED | |  |
| *Thomasomys baeops* | Not found north of equator | | |
| *Thomasomys bombycinus* | Less than 20 occurrences | | |
| *Thomomys bottae* | INCLUDED | |  |
| *Thomomys bulbivorus* | Less than 20 occurrences | | |
| *Thomasomys cinereiventer* | INCLUDED | |  |
| *Thomasomys cinereus* | Not found north of equator | | |
| *Thomomys clusius* | Less than 20 occurrences | | |
| *Thomasomys daphne* | Not found north of equator | | |
| *Thomasomys eleusis* | Not found north of equator | | |
| *Thomasomys erro* | Not found north of equator | | |
| *Thomasomys gracilis* | Not found north of equator | | |
| *Thomasomys hylophilus* | INCLUDED | |  |
| *Thomomys idahoensis* | INCLUDED | |  |
| *Thomasomys incanus* | Not found north of equator | | |
| *Thomasomys ischyrus* | Not found north of equator | | |
| *Thomasomys kalinowskii* | Not found north of equator | | |
| *Thomasomys ladewi* | Not found north of equator | | |
| *Thomasomys laniger* | INCLUDED | |  |
| *Thomasomys macrotis* | Not found north of equator | | |
| *Thomomys mazama* | INCLUDED | |  |
| *Thomasomys monochromos* | Less than 20 occurrences | | |
| *Thomomys monticola* | Less than 20 occurrences | | |
| *Thomasomys niveipes* | Less than 20 occurrences | | |
| *Thomasomys notatus* | Not found north of equator | | |
| *Thomasomys onkiro* | Not found north of equator | | |
| *Thomasomys oreas* | Not found north of equator | | |
| *Thomasomys paramorum* | Not found north of equator | | |
| *Thomasomys pyrrhonotus* | Not found north of equator | | |
| *Thomasomys rhoadsi* | Not found north of equator | | |
| *Thomasomys rosalinda* | Not found north of equator | | |
| *Thomasomys silvestris* | Not found north of equator | | |
| *Thomasomys taczanowskii* | Not found north of equator | | |
| *Thomomys talpoides* | INCLUDED | |  |
| *Thomomys townsendii* | INCLUDED | |  |
| *Thomasomys ucucha* | Not found north of equator | | |
| *Thomomys umbrinus* | INCLUDED | |  |
| *Thomasomys vestitus* | Less than 20 occurrences | | |
| *Thrichomys apereoides* | Not found north of equator | | |
| *Thylamys cinderella* | Not found north of equator | | |
| *Thylamys elegans* | Not found north of equator | | |
| *Thylamys macrurus* | Not found north of equator | | |
| *Thylamys pallidior* | Not found north of equator | | |
| *Thylamys pusillus* | Not found north of equator | | |
| *Thylamys tatei* | Not found north of equator | | |
| *Thylamys velutinus* | Not found north of equator | | |
| *Thylamys venustus* | Not found north of equator | | |
| *Thyroptera discifera* | INCLUDED | |  |
| *Thyroptera lavali* | Not found north of equator | | |
| *Thyroptera tricolor* | INCLUDED | |  |
| *Tlacuatzin canescens* | INCLUDED | |  |
| *Tolypeutes matacus* | Not found north of equator | | |
| *Tolypeutes tricinctus* | Not found north of equator | | |
| *Tomopeas ravus* | Not found north of equator | | |
| *Tonatia bidens* | INCLUDED | |  |
| *Tonatia saurophila* | INCLUDED | |  |
| *Trachops cirrhosus* | INCLUDED | |  |
| *Tremarctos ornatus* | INCLUDED | |  |
| *Trinomys albispinus* | Not found north of equator | | |
| *Trinomys dimidiatus* | Not found north of equator | | |
| *Trinomys gratiosus* | Not found north of equator | | |
| *Trinomys iheringi* | Not found north of equator | | |
| *Trinomys myosuros* | Not found north of equator | | |
| *Trinycteris nicefori* | INCLUDED | |  |
| *Trinomys paratus* | Not found north of equator | | |
| *Trinomys setosus* | Not found north of equator | | |
| *Tylomys bullaris* | Less than 20 occurrences | | |
| *Tylomys fulviventer* | Less than 20 occurrences | | |
| *Tylomys mirae* | INCLUDED | |  |
| *Tylomys nudicaudus* | INCLUDED | |  |
| *Tylomys panamensis* | Less than 20 occurrences | | |
| *Tylomys tumbalensis* | Less than 20 occurrences | | |
| *Tylomys watsoni* | Less than 20 occurrences | | |
| *Tympanoctomys barrerae* | Not found north of equator | | |
| *Urocyon cinereoargenteus* | INCLUDED | |  |
| *Urocyon littoralis* | Less than 20 occurrences | | |
| *Uroderma bilobatum* | INCLUDED | |  |
| *Uroderma magnirostrum* | INCLUDED | |  |
| *Ursus americanus* | INCLUDED | |  |
| *Ursus arctos* | INCLUDED | |  |
| *Vampyriscus bidens* | INCLUDED | |  |
| *Vampyriscus brocki* | Less than 20 occurrences | | |
| *Vampyrodes caraccioli* | INCLUDED | |  |
| *Vampyressa melissa* | INCLUDED | |  |
| *Vampyressa nymphaea* | INCLUDED | |  |
| *Vampyressa pusilla* | Not found north of equator | | |
| *Vampyrum spectrum* | INCLUDED | |  |
| *Vampyressa thyone* | INCLUDED | |  |
| *Vicugna vicugna* | Not found north of equator | | |
| *Vulpes lagopus* | INCLUDED | |  |
| *Vulpes macrotis* | INCLUDED | |  |
| *Vulpes velox* | INCLUDED | |  |
| *Vulpes vulpes* | INCLUDED | |  |
| *Wiedomys pyrrhorhinos* | Not found north of equator | | |
| *Wilfredomys oenax* | Not found north of equator | | |
| *Xenomys nelsoni* | Less than 20 occurrences | | |
| *Zaedyus pichiy* | Not found north of equator | | |
| *Zapus hudsonius* | INCLUDED | |  |
| *Zapus princeps* | INCLUDED | |  |
| *Zapus trinotatus* | INCLUDED | |  |
| *Zygodontomys brevicauda* | INCLUDED | |  |
| *Zygodontomys brunneus* | Less than 20 occurrences | | |
| *Zygogeomys trichopus* | Less than 20 occurrences | | |
|  |  |  |  |
