# Supplementary material for: Mean Annual Precipitation Explains Spatiotemporal Patterns of Cenozoic Mammal Beta Diversity and Latitudinal Diversity Gradients in North America
Source: PLoS One. 2014 Sep 9;9(9):e106499. doi: 10.1371/journal.pone.0106499 (PMC4159275; doi:10.1371/journal.pone.0106499)
Supplement: Appendix S1 — Sources for the majority of mammal occurrence data downloaded from the Fossilworks database. (DOCX) [file pone.0106499.s005.docx]

Appendix S1. Sources for the majority of mammal occurrence data downloaded from the Fossilworks database.

X. Wang, R. H. Tedford, and B. E. Taylor. 1999. Phylogenetic systematics of the Borophaginae (Carnivora: Canidae). *Bulletin of the American Museum of Natural History* **243**:1-392 [206 collections, 668 occurrences]

A. F. Pajak, III, E. Scott, and C. J. Bell. 1996. *PaleoBios* **17(2-4)**:28-49 [118 collections, 315 occurrences]

L. M. Abraczinskas. 1993. Pleistocene proboscidean sites in Michigan: New records and an update on published sites. *Michigan Academician* **25(4)**:443-490 [186 collections, 189 occurrences]

J. R. Macdonald. 1970. Review of the Miocene Wounded Knee faunas of southwestern South Dakota. *Bulletin of the Los Angeles County Museum of Natural History, Science* **8**:165-82 [113 collections, 195 occurrences]

M. L. Cassiliano. 1999. Biostratigraphy of Blancan and Irvingtonian mammals in the Fish Creek-Vallecito Creek section, southern California, and a review of the Blancan-Irvingtonian boundary. *Journal of Vertebrate Paleontology* **19(1)**:169-186 [48 collections, 420 occurrences]

C. B. Schultz and C. H. Falkenbach. 1968. *Bulletin of the American Museum of Natural History* **139** [135 collections, 137 occurrences]

X. Wang. 1994. Phylogenetic systematics of the Hesperocyoninae (Carnivora: Canidae). *Bulletin of the American Museum of Natural History* **221**:1-207 [76 collections, 196 occurrences]

M. R. Voorhies. 1990. In T. C. Gustavson (ed.), *Bureau of Economic Geology Guidebook* [18 collections, 683 occurrences]

G. T. James. 1963. Paleontology and nonmarine stratigraphy of the Cuyama Valley badlands, California; Part 1, Geology, faunal interpretations, and systematic descriptions of Chiroptera, Insectivora, and Rodentia. *University of California Publications in Geological Sciences* **45**:1-154 [60 collections, 109 occurrences]

G. T. Jefferson. 1991. A catalogue of late Quaternary vertebrates from California. Part two, mammals. *Natural History Museum of Los Angeles County Technical Report* **7**:1-129 [37 collections, 165 occurrences]

M. F. Skinner, S. M. Skinner, and R. J. Gooris. 1977. Stratigraphy and biostratigraphy of late Cenozoic deposits in central Sioux County, western Nebraska.*Bulletin of the American Museum of Natural History* **158(5)**:263-370 [44 collections, 105 occurrences]

R. Eshelman and F. Grady. 1986. Quaternary vertebrate localities of Virginia and their avian and mammalian fauna. *Virginia Division of Mineral Resources, Publication* **75**:43-70 [28 collections, 163 occurrences]

E. H. Lindsay. 1972. Small mammal fossils from the Barstow Formation, California. *University of California Publications in Geological Sciences* **93**:1-104 [35 collections, 128 occurrences]

P. R. Bjork. 1970. *Transactions of the American Philosophical Society* **60(7)** [56 collections, 79 occurrences]

S. D. Webb. 1974. Chronology of Florida Pleistocene mammals. In S. D. Webb (ed.), *Pleistocene Mammals of Florida* 5-31 [17 collections, 254 occurrences]

J. A. Holman, D. C. Fisher, and R. O. Kapp. 1986. Recent discoveries of fossil vertebrates in the lower peninsula of Michigan. *Michigan Academician***18(3)**:431-463 [56 collections, 60 occurrences]

B. E. Bailey. 2004. Biostratigraphy and biochronology of early Arikareean through late Hemingfordian small mammal faunas from the Nebraska Panhandle and adjacent areas. *Paludicola* **4(3)**:81-113 [11 collections, 250 occurrences]

S. D. Webb. 1969. *University of California Publications in Geological Sciences* **78** [22 collections, 110 occurrences]

E. H. Lindsay and N. T. Tessman. 1974. *Journal of the Arizona Academy of Sciences* **9** [24 collections, 96 occurrences]

G. S. Morgan and R. C. Hulbert, Jr. 1995. *Bulletin of the Florida Museum of Natural History* **37(1)** [12 collections, 187 occurrences]
